# Regulation of claudin/zonula occludens-1 complexes by hetero-claudin interactions

**DOI:** 10.1038/ncomms12276

**Published:** 2016-07-25

**Authors:** Barbara Schlingmann, Christian E. Overgaard, Samuel A. Molina, K. Sabrina Lynn, Leslie A. Mitchell, StevenClaude Dorsainvil White, Alexa L. Mattheyses, David M. Guidot, Christopher T. Capaldo, Michael Koval

**Affiliations:** 1 Division of Pulmonary, Allergy, Critical Care and Sleep Medicine, Department of Medicine, Emory University, 205 Whitehead Building, 615 Michael Street, Atlanta, Georgia 30322, USA; 2 Emory Alcohol and Lung Biology Center, Emory University, Atlanta, Georgia 30322, USA; 3 Department of Cell Biology, Emory University, Atlanta, Georgia 30322, USA; 4 Atlanta Veterans Affairs Medical Center, Decatur, Georgia 30033, USA; 5 Department of Pathology; Emory University School of Medicine, Atlanta, GA 30322, USA

## Abstract

Claudins are tetraspan transmembrane tight-junction proteins that regulate epithelial barriers. In the distal airspaces of the lung, alveolar epithelial tight junctions are crucial to regulate airspace fluid. Chronic alcohol abuse weakens alveolar tight junctions, priming the lung for acute respiratory distress syndrome, a frequently lethal condition caused by airspace flooding. Here we demonstrate that in response to alcohol, increased claudin-5 paradoxically accompanies an increase in paracellular leak and rearrangement of alveolar tight junctions. Claudin-5 is necessary and sufficient to diminish alveolar epithelial barrier function by impairing the ability of claudin-18 to interact with a scaffold protein, zonula occludens 1 (ZO-1), demonstrating that one claudin affects the ability of another claudin to interact with the tight-junction scaffold. Critically, a claudin-5 peptide mimetic reverses the deleterious effects of alcohol on alveolar barrier function. Thus, claudin controlled claudin-scaffold protein interactions are a novel target to regulate tight-junction permeability.

## Abstract (web summary)

Alcohol abuse is a risk factor for acute respiratory distress syndrome, flooding of the lungs due to compromised barrier function. Here the authors report that alcohol upregulates claudin-5 that is then recruited to tight junctions in alveolar epithelial cells, causing the displacement of claudin-18 from ZO-1 and diminished barrier function.

## Graphical abstract

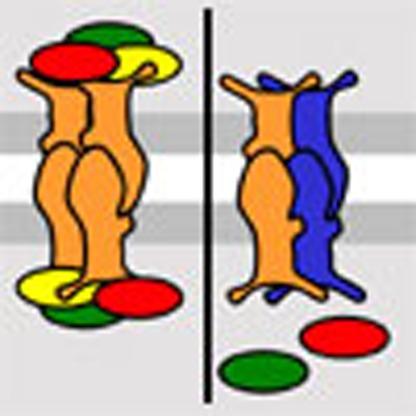

There are ample clinical data demonstrating that alcoholics are at increased risk of acute respiratory distress syndrome compared with non-alcoholic patients due to a failure in lung fluid clearance leading to airspace flooding, which critically impairs gas exchange across the alveolar epithelium^1^,^2^. Dietary alcohol significantly impairs alveolar epithelial cell (AEC) tight junctions that are required to provide a barrier between fluid-filled tissues and the airspace^3^. However, the molecular basis for the effects of alcohol on alveolar epithelial tight junctions is not well understood. Here we have used isolated primary rat AECs that differentiate into a model type-I monolayer that enables barrier function to be studied at a molecular level. Rats fed dietary alcohol for 8 weeks provide an animal model system that faithfully recapitulates the pathologic consequences of chronic alcohol ingestion on lung barrier function^4^,^5^. Moreover, primary cells derived from alcohol-fed rats (alcohol-exposed AECs) have impaired barrier function that persists *in vitro*, as compared with AECs isolated from animals fed an isocaloric control diet.

Thus, we studied cultured, polarized AECs derived from control and alcohol-fed animals as a model system that reflects the behaviour of these cells *in vivo* in forming the alveolar barrier. AECs from alcohol-fed animals have significant changes in tight-junction protein expression that are associated with a decrease in epithelial barrier function. Among these changes is an increase in claudin-5 expression. By molecular manipulation of AECs we find that claudin-5 is both necessary and sufficient to disrupt AEC tight junctions. Increased claudin-5 expression induces the formation of claudin-containing structures perpendicular to the axis of the cell–cell interface (tight-junction spikes) that are active sites of vesicle budding and fusion. The appearance of tight-junction spikes correlates with increased paracellular leak between AECs. Using several complementary approaches, including super-resolution microscopy and the proximity ligation assay (PLA), we find that claudin-5 interacted with claudin-18, and that this decreases the ability of claudin-18 to productively interact with zonula occludens 1 (ZO-1). This provides the first example of one claudin affecting the ability of another claudin to interact with the tight-junction scaffold. This mechanism is targetable using a claudin-5 mimetic peptide, suggesting a potential therapeutic approach to promote alveolar barrier function.

## Results

### Chronic alcohol alters lung tight-junction permeability

**Figure 1:**
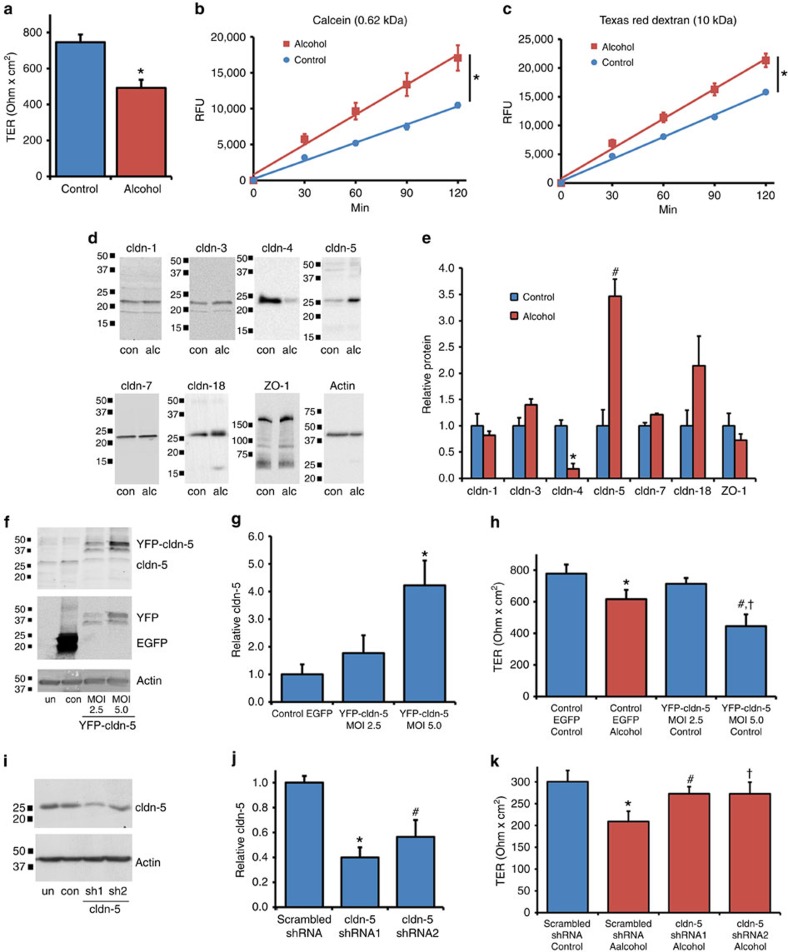
Alcohol dependent upregulation of claudin-5 is necessary and sufficient to impair alveolar barrier function. AECs from alcohol fed rats and controls were cultured on Transwell permeable supports and then transepithelial resistance (TER) (**a**) and dye flux with calcein (**b**) and Texas Red Dextran (**c**) were measured. Alcohol-exposed AECs showed a significantly lower TER (*n*=6, *P<0.001, unpaired two-tailed *t*-test) as well as significantly higher calcein (*n*=3, *P<0.001, two way ANOVA with Bonferroni multiple comparisons test) and Texas Red Dextran permeability (*n*=3, *P<0.001, two way ANOVA) versus cells from control fed rats. (**d**,**e**) By immunoblot, alcohol exposure significantly decreased claudin-4 expression (*n*=3, *P=0.002, *t*-test) and significantly increased claudin-5 expression by AECs (*n*=3, ^#^*P*=0.005, *t*-test). (**f**–**h**) Control AECs were transduced with adenovector YFP-claudin-5 at MOI of 2.5 or 5 or with EGFP adenovector at MOI of 5 as a control. The EGFP/EYFP doublet has been seen by others^60^,^61^ and has no bearing on our results since untagged claudin-5 has a comparable effect on AECs (Supplementary Fig. 5). (**f**,**g**) YFP-claudin-5 at MOI of 5 significantly increased claudin-5 expression (*n*=3, *P=0.022, one way ANOVA with Tukey multiple comparisons test) and (**h**) decreased TER (*n*=3, ^#^*P*=0.0005 versus EGFP transduced control AECs; ^†^*P*=0.028 versus EGFP transduced alcohol exposed cells, one way ANOVA). In (**h**), TER of alcohol exposed cells was significantly lower than comparable control cells (*n*=3, *P=0.036, one way ANOVA). (**i**–**k**) Claudin-5 protein expression in alcohol-exposed AECs was depleted using a lentiviral system delivering shRNA targeting claudin-5 or control scrambled shRNAs. (**i**,**j**) Claudin-5 was significantly depleted by specific shRNAs versus scrambled shRNA treated cells (*n*=4, *P=0.006, ^#^*P*=0.036, one way ANOVA). (**k**) decreased claudin-5 expression in alcohol-exposed cells significantly increased TER as compared with cells transduced with scrambled shRNAs (*n*=4, ^#^*P*<0.001, ^†^*P*<0.001, one way ANOVA). TER of cells from alcohol exposed cells treated with shRNA was significantly lower than comparable control cells (*n*=4, *P<0.001, one way ANOVA). All quantitative data represents average ±s.e.m.

The difference between AECs isolated from control- and alcohol-fed animals (alcohol-exposed AECs) is demonstrated in Fig. 1a–c, using two different measures of barrier function: transepithelial resistance (TER) and paracellular flux to soluble tracer molecules. Consistent with an increase in paracellular leak, alcohol-exposed AECs had significantly decreased TER and showed increased flux of both calcein (0.62 kDa) and Texas Red Dextran (10 kDa). Thus, alcohol exposure has a deleterious effect on AEC tight junctions, consistent with previous reports^4^,^6^.

As claudins are central to the regulation of tight-junction permeability^7^,^8^,^9^, claudin protein composition of control- and alcohol-exposed AECs cultured on Transwell-permeable supports was examined by immunoblotting. The decrease in AEC barrier function induced by alcohol correlated with decreased claudin-4 protein (Fig. 1d,e). Claudin-1, claudin-3 and claudin-7 were unaffected. However, AEC-associated claudins did not simply decrease in response to alcohol. Instead, claudin-5 was significantly increased in alcohol-exposed AECs as compared with control AECs (Fig. 1d,e), consistent with previous analysis of freshly isolated type II cells and AECs cultured on tissue culture plastic^10^. There also was a trend towards increased claudin-18 in alcohol-exposed AECs as compared with control AECs (*P*=0.15, *n*=3, unpaired two-tailed *t*-test). As there was increased paracellular leak accompanying increased claudin-5 expression, we examined the effects of claudin remodelling in response to alcohol, to determine whether this had a destabilizing effect on tight junctions.

### Increased claudin-5 causes increased paracellular leak

In particular, increased claudin-5 expression by lung epithelial cells has previously been associated with an increase in paracellular leak by AECs^11^. To confirm whether increased claudin-5 was sufficient to increase paracellular leak, we examined the dose response of increased yellow fluorescent protein (YFP)–claudin-5 expression using an adenovector to transduce primary AECs. A fourfold increase in claudin-5 expression ((YFP–claudin-5+claudin-5)/claudin-5) significantly decreased TER (Fig. 1f–h) and increased paracellular flux (Supplementary Fig. 1a,b). Critically, this level of YFP–claudin-5 expression is in the physiologic range, comparable to the increase in endogenous AEC claudin-5 expression induced by alcohol (Fig. 1e). In the converse experiment, lentiviral short hairpin RNA (shRNA) constructs were used to decrease claudin-5 expression (Supplementary Table 1). As shown in Fig. 1i–k, using shRNA to decrease claudin-5 expression by AECs from alcohol-fed rats caused a significant increase in TER and also decreased paracellular flux (Supplementary Fig. 1c,d).

As claudin-4 decreased in response to dietary alcohol, it could also have a negative impact on AEC barrier function in combination with increased claudin-5. Thus, we examined whether increased claudin-4 could rescue the effects of alcohol on AECs. As shown in Supplementary Fig. 2a, alcohol-exposed AECs transduced with CFP-claudin-4 had only a partial increase in TER compared with control AECs. Moreover, the effects of increased claudin-4 were antagonized by a concurrent transduction with YFP–claudin-5. The fact that claudin-5 countered the ability of claudin-4 to promote paracellular barrier function suggests that these claudins are directly interacting. Formation of complexes containing native claudin-4 and native claudin-5 was confirmed by co-immunopurification analysis of AECs (Supplementary Fig. 2b). We also observed using co-immunopurification that native claudin-5 directly interacts with native claudin-18 and ZO-1 (Supplementary Fig. 2g). These data further support the hypothesis that increased claudin-5 has a deleterious and dominant effect on other claudins and thereby impairs AEC barrier function.

### Tight-junction spikes are associated with barrier disruption

**Figure 2:**
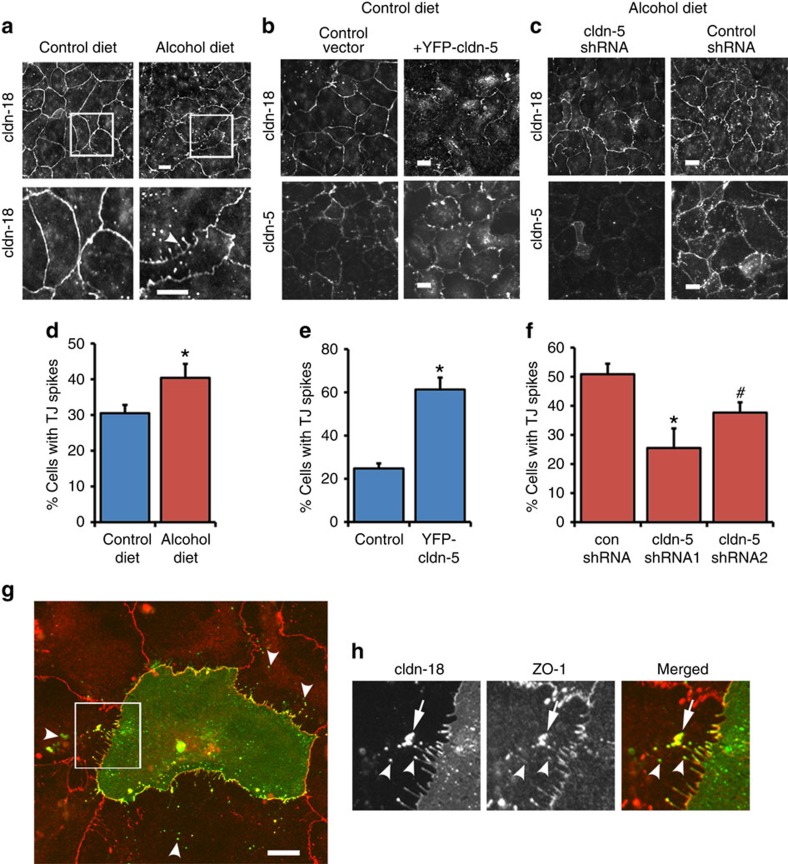
Increased claudin-5 expression enhances the formation of tight junction spikes. AECs isolated from alcohol or control fed rats were cultured for 5–7 days on transwell permeable supports and immunolabeled for claudin-18. (**a**) Cells from alcohol fed rats showed enhancement of tight junction spikes, that are claudin-18 projections perpendicular to the cell-cell interface (d; arrowhead). Square regions in the top panels correspond to magnified images in below (Bar, 10 μm). (**b**) Control AECs transduced with YFP-claudin-5 increased the appearance of tight junction spikes as determined by labeling for cldn-18 or cldn-5 (Bar, 10 μm). (**c**) Alcohol-exposed AECs transduced with claudin-5 shRNA had a decrease in tight junction spikes (Bar, 10 μm). (**d**–**f**) Quantification of the % of cells containing 3 or more tight junction spikes oriented towards the nucleus demonstrated that alcohol exposed and YFP-claudin-5 transduced AECs had significantly more spikes than comparable controls. (**d**) Control versus alcohol: *n*=11 fields, *P=0.035, unpaired two-tailed *t*-test. (**e**) EGFP versus YFP-claudin-5: *n*=11 fields, *P<0.001, unpaired two-tailed *t*-test. (**f**) Alcohol exposed AECs transduced with claudin-5 shRNA1 had significantly fewer spikes than cells treated with control shRNA (*n*=5 fields, *P=0.011, one way ANOVA with Tukey multiple comparisons test). Cells treated with shRNA2 showed a trend towards decreased spikes (*n*=5, ^#^*P*=0.18, one way ANOVA with Tukey multiple comparisons test) (**g**). Control AECs were partially transfected with YFP-claudin-18 then fixed and immunolabeled for ZO-1. YFP-claudin-18 expressing cells adjacent to untransfected cells showed uptake of YFP-claudin-18 in intracellular vesicles (arrows, Bar, 10 μm). (**h**) Magnified images corresponding to the square region in (**g**) showing spike associated claudin-18 internalized into adjacent cells. Arrowheads show areas where claudin-18 does not co-localize with ZO-1. The arrow indicates a structure where YFP-claudin-18 and ZO-1 co-localize. All quantitative data represents average ±s.e.m.

As revealed by immunofluorescence microscopy of claudin-18 (Fig. 2a), AECs from alcohol-fed rats have changes in tight-junction morphology, most notably increased formation of tight-junction spikes (Fig. 2d), which are actin-associated structures perpendicular to the axis of the cell–cell interface that correlate with an increase in paracellular leak^4^,^5^. Normal AECs transduced to express increased claudin-5 also showed an increase in claudin-18 containing spikes, comparable to the effect of alcohol on tight-junction morphology (Fig. 2b,e). Morphologic disruption of tight junctions was not restricted to claudin-18, as claudin-5 (Fig. 2b) and ZO-1 (Supplementary Fig. 3j–l) were also impaired in YFP–claudin-5-transduced AECs. To determine whether ZO-1 disruption was specifically linked to increased claudin-5, we examined the effect of increased YFP–claudin-3 on ZO-1 localization by AECs and found there was little effect on tight-junction morphology based on localization of claudin-18 (Supplementary Fig. 3g–i) or ZO-1 (Supplementary Fig. 3m–o)^12^. In a complementary experiment, we determined whether the ability of alcohol to induce formation of tight-junction spikes was antagonized by depleting claudin-5 using shRNA. As shown in Fig. 2c,f, this was the case for two different specific claudin-5 shRNAs. Thus, claudin-5 was necessary and sufficient to enhance formation of tight-junction spikes.

To rule out an effect of YFP–claudin-5 expression on levels of other key AEC tight-junction proteins, we examined expression of claudin-1, claudin-3, claudin-4, endogenous claudin-5, claudin-7, claudin-18 and ZO-1 by AECs transduced with YFP–claudin-5. As shown in Supplementary Fig. 4, YFP–claudin-5 expression had little effect on total levels of these tight-junction proteins in AECs. We also wanted to ensure that the effects of YFP–claudin-5 on AECs were not due to the amino-terminal YFP tag. AECs transduced with untagged claudin-5 faithfully recapitulated the effects of alcohol on these cells, namely increased formation of tight-junction spikes and impaired barrier function (Supplementary Fig. 5).

Although tight-junction spikes correlated with diminished paracellular barrier function, how spikes were mechanistically linked to paracellular leak was not known. We hypothesized that spikes represented areas of enhanced tight-junction protein reorganization, which is known to increase paracellular leak. To address this, we used AECs expressing YFP–claudin-18 that were adjacent to untransfected AECs (Fig. 2g,h). It is noteworthy that YFP–claudin-18 acts to label tight-junction spikes in live cells and did not induce formation of spikes in a manner comparable to claudin-5. Spike-associated YFP–claudin-18 was found to be internalized by neighbouring non-transduced cells, suggesting that the adjacent cells internalized claudin-18 from neighbouring cells. Moreover, co-localization of ZO-1 to YFP–claudin-18 was variable, as there were readily visualized YFP–claudin-18 structures that lacked co-localization with ZO-1 (Fig. 2h; arrowheads), although claudin-18 and ZO-1 did co-localize in other spike-associated structures (Fig. 2h; arrows).

**Figure 3:**
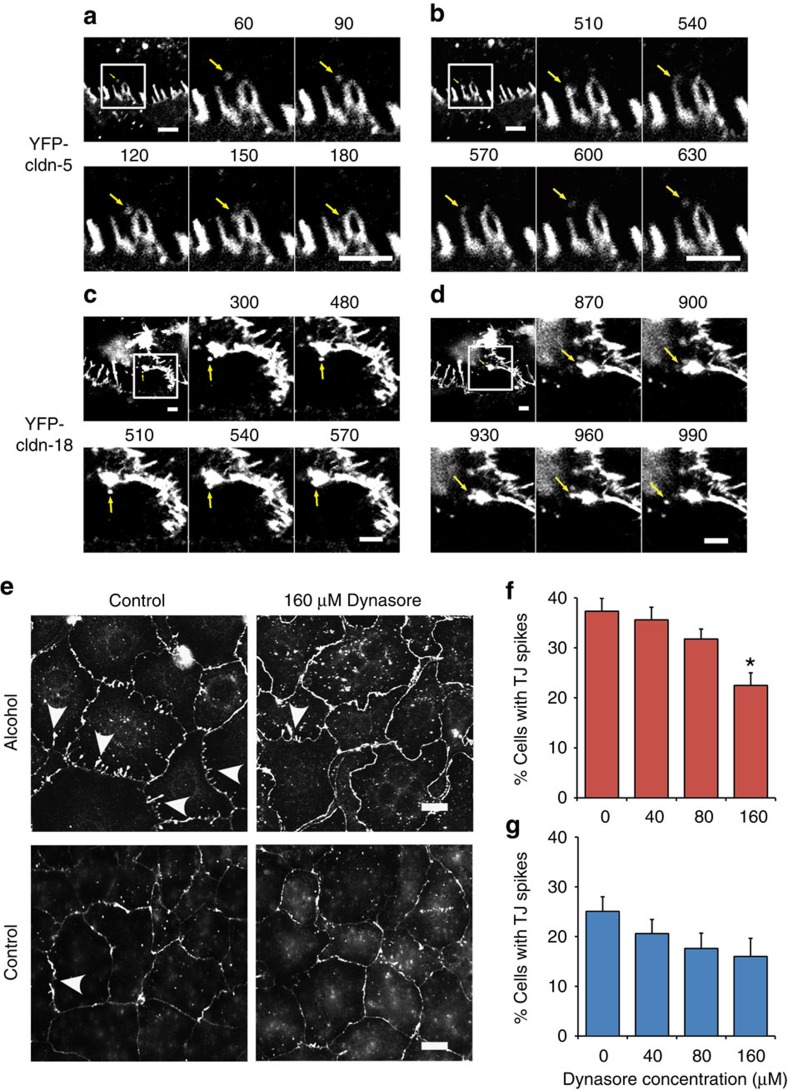
Claudin-containing vesicles bud from and fuse with tight junction spikes. (**a**–**d**) Live cell imaging was performed with alcohol exposed AECs transduced with Adenovirus encoding either YFP-claudin-5 (**a**,**b**) or YFP-claudin-18 (**c**,**d**). Shown are still images from videos acquired over a 20 min time period with a frame capture of 30 s intervals. Labeled vesicles containing YFP-claudin-5 or YFP-claudin-18 were found to both fuse to (**a**,**c**) and bud from (**b**,**d**) tight junction spikes, demonstrating that these are dynamic structures. The top left panel in each series is a lower magnification image, the square region represents the time series, which is time stamped in seconds. Bar, 5 μm. (**e**) Cells from alcohol fed or control fed rats were cultured for 7 days and then treated with either DMSO vehicle control or the dynamin inhibitor Dynasore at varying concentrations for 4 h at 37 °C in serum free media. The cells were then fixed and immunolabeled for claudin-18. Representative images show vehicle-treated and 160 μM Dynasore treated cells. Arrowheads show tight junction spikes. Bar, 10 μm. (**f**,**g**) Quantification of the % cells containing 3 or more tight junction spikes oriented towards the nucleus demonstrated that 160 μM Dynasore significantly decreased the number of cells from alcohol fed rats containing spikes (*n*=8-9 fields, *P=0.002, one way ANOVA with Tukey multiple comparisons test) (**f**). Dynasore did not have a significant effect on spike formation by control cells (**g**). All quantitative data represents average±s.e.m.

To further characterize the behaviour of claudins associated with tight-junction spikes, we used live-cell imaging microscopy of alcohol-exposed AECs transduced to express either YFP–claudin-5 (Fig. 3a,b and Supplementary Video 1) or YFP–claudin-18 (Fig. 3c,d and Supplementary Video 2), which revealed the dynamic nature of tight-junction spikes. Specifically claudin-labelled vesicles were found to both fuse with (Fig. 3a,c) and bud from (Fig. 3b,d) tight-junction spikes. To further confirm that spikes were sites of active claudin vesicle formation and fusion^13^, we examined the effects of the dynamin inhibitor Dynasore^14^ on spike formation by alcohol-exposed AECs. Consistent with this, treatment with Dynasore at 160 μM for 4 h caused a significant decrease in the number of cells with tight-junction spikes (Fig. 3e,f) comparable to the number of cells containing spikes observed for untreated control AECs (Fig. 3e,g). Dynasore-treated cells also showed an increase in punctate YFP–claudin-18 labelling, which probably represents secretory and endocytic vesicles that are inhibited from fusing with target intracellular membranes by Dynasore. As an increase in tight-junction spikes correlated with decreased barrier function, these data suggest that increased vesicle-mediated trafficking of claudins both into and out of tight junctions contributes to paracellular leak in response to alcohol.

### Claudin-5 alters interactions between claudin-18 and ZO-1

**Figure 4:**
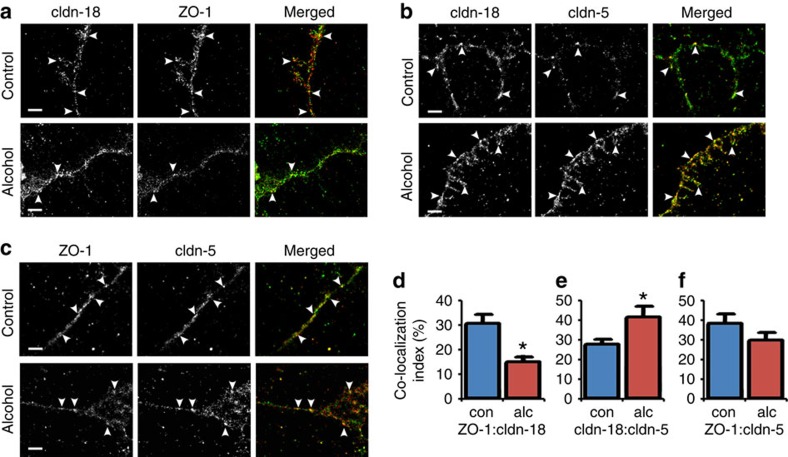
Claudin-5 induced by alcohol decreases ZO-1:claudin-18 co-localization as determined by super-resolution microscopy. (**a**–**c**) AECs isolated from alcohol (alc) or control (con) fed rats were cultured, immunolabeled and imaged by STORM. Cells were double-labeled for claudin-18 and ZO-1 (**a**), claudin-5 and claudin-18 (**b**) or claudin-5 and ZO-1 (**c**). Images were analyzed for protein co-localization (**d**–**f**). Alcohol exposed AECs showed a reduction in the co-localization between claudin-18 and ZO-1 and an increase in co-localization between claudin-18 and claudin-5. Co-localization between claudin-5 and ZO-1 was comparable for both control and alcohol exposed cells. Arrowheads denote areas of co-localization. Bar, 1 μm. (**d**–**f**) Quantification of co-localization using STORM images demonstrated a significant change. In alcohol-exposed AECs there was a significant decrease in ZO-1:claudin-18 (*n*=4 fields (control), *n*=3 fields (alcohol exposed AECs),*P=0.014, unpaired two-tailed *t*-test) (**d**) which correlated with a significant increase in claudin-18:claudin-5 co-localization (*n*=3 fields, *P=0.039, unpaired two-tailed *t*-test) (**e**). ZO-1:claudin-5 co-localization was unchanged (*n*=4 fields, unpaired two-tailed *t*-test) (**f**). Data in (**d**–**f**) represent average±s.e.m.

As tight junctions are multi-protein complexes, paracellular barrier function requires coordinating heterologous interactions between tight-junction proteins. In intact cell junctions, protein–protein interactions are reflected by co-localization of two or more proteins in the same intracellular location when resolved at sufficient resolution. To understand how alcohol-induced changes affect tight junctions at a molecular level, we examined AECs isolated from control- and alcohol-fed rats by a form of super-resolution immunofluorescence microscopy, stochastic optical reconstruction microscopy (STORM), which has an *X*–*Y* resolution down to 20 nm (Fig. 4 and Supplementary Fig. 6)^15^,^16^. By the nature of the technique, STORM provides images that are composed of point densities, resulting in a particulate image at high magnification. We noticed that STORM images obtained using the same labelling and imaging conditions appeared to have differences in the size of particulate clusters when comparing control versus alcohol-exposed AECs. Thus, we quantified the distribution of particulate clusters (Supplementary Fig. 7). STORM imaging of normal AECs showed that claudin-18, claudin-5 and ZO-1 clusters had median areas of 1,240, 1,410 and 1,590 nm^2^, respectively (Supplementary Fig. 7g–i). By contrast, alcohol-exposed AECs had claudin-18, claudin-5 and ZO-1 clusters with median areas of 1,410, 1,000 and 1,120 nm^2^, respectively. The alcohol-induced decrease in median cluster size for claudin-5 and ZO-1 was significant, as determined by Mann–Whitney *U*-test; however, Claudin-18 cluster size was statistically unchanged. As these images were obtained using the same labelling and imaging conditions, the change in claudin-5 and ZO-1 cluster size induced by alcohol is likely to reflect tight-junction re-organization in response to alcohol, despite the inability to assign a specific physiologic correlate to particulate clusters. As shown in Fig. 4 and Supplementary Fig. 6, STORM images of AEC tight junctions showed a predominant linear intercellular complex with some projections and limited meshwork architecture. Some images also showed tight-junction spikes. This contrasts with the super-resolution images obtained by Kauffmann *et al.*^17^ using a comparable technology (spectral position determination microscopy) to analyse claudin-transfected HEK293 cells expressing claudin-3 or claudin-5 at levels optimized to form a native-equivalent junctional meshwork on the apical surface. Nonetheless, it was not surprising that STORM analysis of AECs did not show an extensive meshwork, as tight junctions between adjacent type-I AECs *in situ* were shown to have a fairly limited architecture^18^,^19^. Moreover, STORM images are obtained using the total internal reflection fluorescence mode of illumination and thus any junctional elements perpendicular to the narrow plane of focus would not be revealed using our approach. Here we optimized the STORM-imaging conditions for co-localization analysis between tight-junction proteins as opposed to maximizing imaging resolution.

STORM enabled quantitative differences in co-localization to be measured, as we performed these measurements where cross-talk between the two different channels was minimized (Supplementary Fig. 8). In alcohol-exposed AECs, there was a significant decrease in co-localization between claudin-18 and ZO-1 as compared with control AECs (Fig. 4d). Conversely, there was an increase in co-localization between claudin-18 and claudin-5 in AECs isolated from alcohol-fed rats as compared with controls (Fig. 4e). This reciprocal relationship supports the hypothesis that in response to interacting with claudin-5, claudin-18 dissociates from ZO-1.

**Figure 5:**
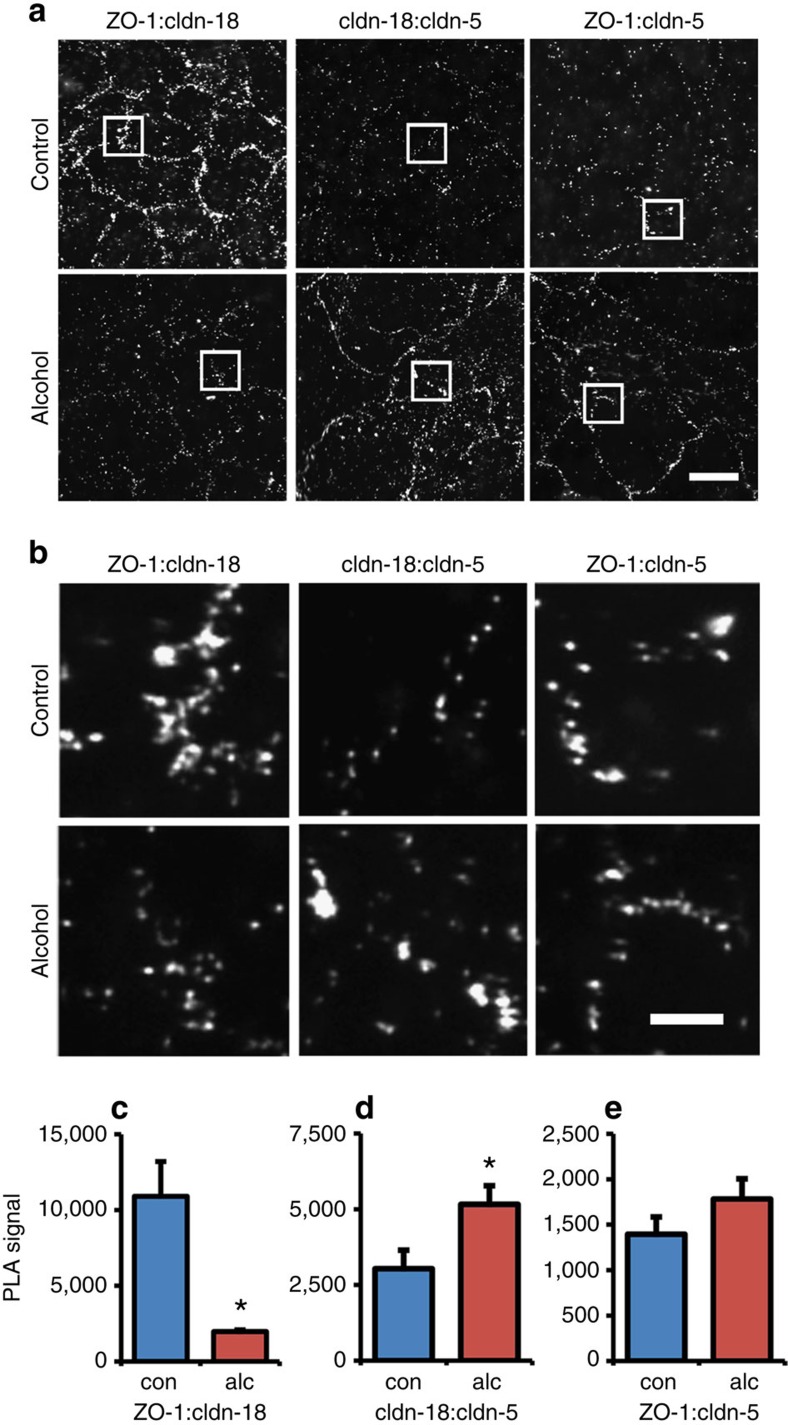
Claudin-5 induced by alcohol decreases ZO-1:claudin-18 co-localization as determined by proximity ligation assay. (**a**,**b**) AECs isolated from alcohol (alc) or control (con) fed rats were cultured, immunolabeled and analyzed using the proximity ligation assay (PLA). Cells were PLA-labeled for claudin-18 and ZO-1, claudin-5 and claudin-18 or claudin-5 and ZO-1. Images in (**b**) are magnifications of regions in (**a**) as denoted by the squares. Bar, 20 μm. Negative controls are shown in Supplementary Fig. 10. Alcohol exposed AECs showed a reduction in the co-localization between claudin-18 and ZO-1 and an increase in co-localization between claudin-18 and claudin-5. Co-localization between claudin-5 and ZO-1 was comparable for both control and alcohol exposed cells. (**c**–**e**) Quantification of co-localization using PLA demonstrated a significant change. In alcohol-exposed AECs there was a significant decrease in ZO-1:claudin-18 (*n*=6 fields,*P=0.018, unpaired two-tailed *t*-test) (**c**) which correlated with a significant increase in claudin-18:claudin-5 co-localization (*n*=10 fields, *P=0.026, unpaired two-tailed *t*-test) (**d**). ZO-1:claudin-5 co-localization was unchanged (*n*=6 fields, unpaired two-tailed *t*-test) (**e**). Data in (**c**–**e**) represent average±s.e.m.

To further investigate the alcohol-induced changes in ZO-1:claudin-18 co-localization, we examined AECs using the PLA, which has a resolving power of 30–40 nm^20^,^21^. As shown in Fig. 5 and Supplementary Fig. 9, PLA analysis of claudin-18 and ZO-1 in control AECs gave a robust signal. Negative controls are shown in Supplementary Fig. 10. By contrast, alcohol-exposed AECs had a significantly diminished PLA signal (Fig. 5c). Conversely, claudin-18 and claudin-5 had a PLA signal that was increased in alcohol-exposed AECs as compared with control AECs (Fig. 5d). ZO-1:claudin-5 co-localization was comparable for control and alcohol-exposed AECs, although the PLA signals have a slightly different appearance, because the cluster size for both ZO-1 and claudin-5 is sensitive to alcohol (Supplementary Fig. 7). These results parallel our analysis of the effects of alcohol on claudin-18, claudin-5 and ZO-1 co-localization by STORM (Fig. 4d–f). Thus, two independent approaches demonstrate that ZO-1:claudin-18 proximity was diminished by alcohol and correlated with an increase in claudin-18:claudin-5 proximity.

**Figure 6:**
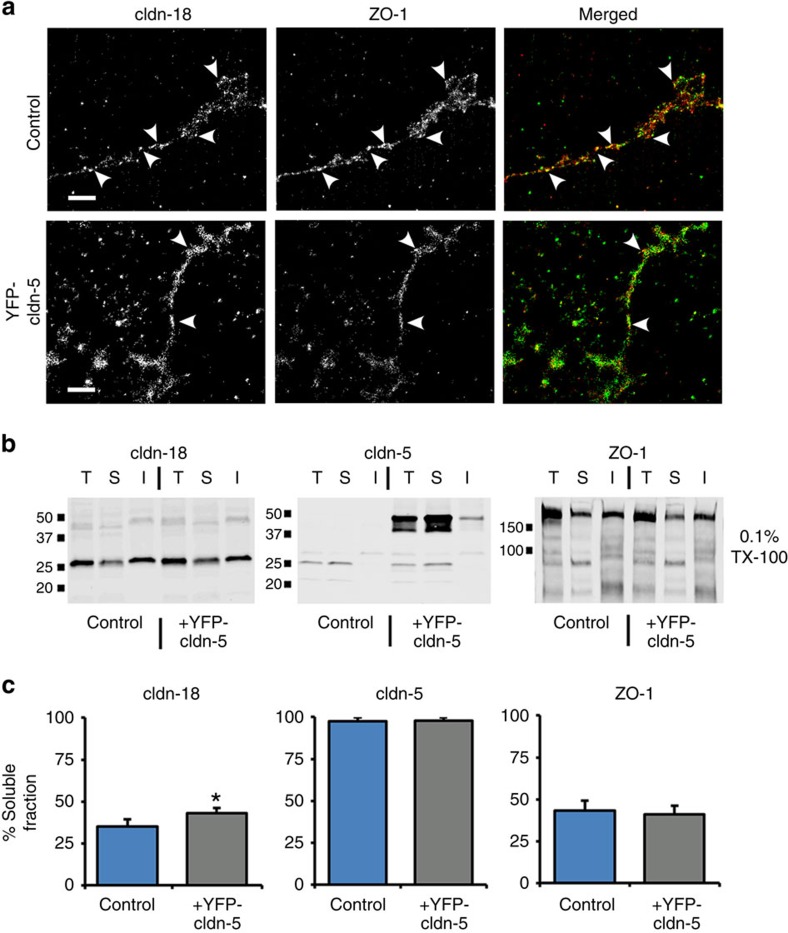
Claudin-5 expression is sufficient to decrease ZO-1:claudin-18 co-localization and increase claudin-18 solubilization. (**a**) Control or YFP-claudin-5 transduced AECs were cultured for 6 days, immunolabeled and then imaged by STORM for claudin-18 and ZO-1. Increased claudin-5 expression decreased the extent of ZO-1:claudin-18 co-localization (see text). Arrowheads show sites of co-localization. Bar, 1 μm. (**b**,**c**) Biochemical analysis of protein insolubility was assessed by a Triton X-100 solubilization assay comparing control AECs to YFP-claudin-5 transduced cells. At 6 days in culture, AECs were harvested and extracted using 0.1% Triton X-100, an aliquot of total protein (T) was set aside and the remainder was centrifuged to separate Triton X-100 soluble (S) and insoluble (I) fractions that were measured by immunoblot for claudin-18, claudin-5 and ZO-1. Quantification of the soluble fraction revealed that YFP-claudin-5 expression significantly increased claudin-18 solubility from 35.2±1.8 to 42.1±0.6 (*n*=3, *P=0.003, unpaired two-tailed *t*-test) while claudin-5 and ZO-1 solubility did not significantly change. All quantitative data represents average±s.e.m.

To determine whether increased claudin-5 was sufficient to decrease association of claudin-18 and ZO-1, we examined AECs transduced with YFP–claudin-5 by STORM (Fig. 6a and Supplementary Fig. 6s–x). As opposed to untransduced AECs, where the co-localization index between claudin-18 and ZO-1 was 30.5±3.6% (mean±s.e.m., *n*=3; Fig. 4d), AECs expressing YFP–claudin-5 had significantly decreased co-localization between claudin-18 and ZO-1 (16.4±3.0%, *n*=3, *P*=0.029, unpaired two-tailed *t*-test) that was comparable to alcohol-exposed AECs (15.2±0.7%, *n*=3, unpaired two-tailed *t*-test; Fig. 4d). The significant drop in co-localization between ZO-1 and claudin-18 is consistent with a decrease in interaction between these two proteins, which we hypothesize would alter the assembly state of claudin-18.

In AECs, both claudin-18 and ZO-1 are highly resistant to Triton X-100 (ref. 12) (Fig. 6b,c), suggesting that ZO-1:claudin-18 complexes are tightly associated with the cytoskeleton^22^. Thus, we examined the effects of increased claudin-5 on the extractability of claudin-18, claudin-5 and ZO-1 by Triton X-100. Consistent with previous measurements, less than ∼35% of claudin-18 can be solubilized by Triton X-100 under conditions where the insoluble fraction primarily reflects proteins incorporated into tight junctions^12^ (Fig. 6c). By contrast, the majority of cell-associated claudin-5 is extractable by Triton X-100.

When AECs were transduced with YFP–claudin-5, the Triton X-100 soluble pool of claudin-18 significantly increased from 35.2±1.8% to 42.1±0.6% (*n*=3; *P*=0.003, unpaired two-tailed *t*-test), representing a 20% increase in claudin-18 solubility (Fig. 6c). However, ZO-1 solubility was unchanged by increased claudin-5 (43.1±6.4% versus 40.4±5.5% (*n*=3)). Instead, the increase in claudin-18 solubility induced by YFP–claudin-5 expression (Fig. 6c) correlated with the decrease in co-localization between claudin-18 and ZO-1 from ∼31% to ∼16% as measured by STORM (see above). This decrease in co-localization suggests that decreased ZO-1:claudin-18 interactions induced by increased claudin-5 are sufficient to destabilize the tight-junctional pool of claudin-18.

### A claudin-5 peptide improves alveolar barrier function

Claudin peptide mimetics corresponding to the extracellular domain^23^,^24^,^25^,^26^,^27^,^28^ and *Clostridium perfringens* enterotoxin variants^29^,^30^ have been successfully used to alter tight-junction permeability and probe for claudin–claudin interactions. This suggested that targeting claudin-5 using an extracellular domain peptide might be an effective approach to improve the barrier function of AECs by inhibiting integration into tight junctions. Analogous to an approach used by Baumgartner *et al.*^27^ to target claudin-3 and claudin-4, we used an acetylated D-amino acid peptide corresponding to the region of the second extracellular (E2) domain directly adjacent to the third transmembrane (TM3) domain of claudin-5 (Ac-EFYDP-NH_2_). The E2/TM3 region is implicated in mediating *cis*–claudin interactions, based on the crystal structure of claudin-15 (ref. 31), as well as functional studies of claudin-3:claudin-5 (ref. 32) and homomeric claudin-5 interactions^33^. In addition, the corresponding region of claudin-18 (NFWMS) is not conserved and this region is sufficiently divergent from the corresponding DFYNP sequence found in other major claudins found in the lung, including claudin-3, -4 and -7. Claudin-1 does have an EFYDP motif; however, it is present at low levels in AECs, suggesting that the Ac-EFYDP-NH_2_ peptide could effectively target claudin-5 and reverse the effects of alcohol on tight junctions.

**Figure 7:**
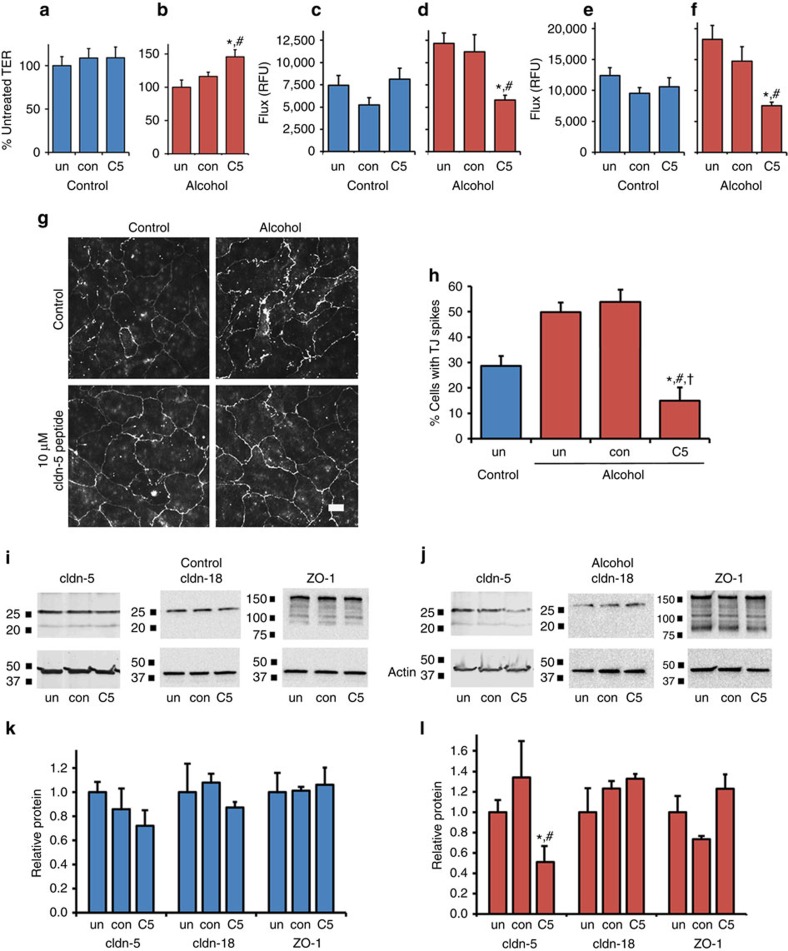
A claudin-5 extracellular domain mimetic increases barrier function of alcohol-exposed AECs. (**a**–**f**) AECs isolated from control (**a**,**c**,**e**) or alcohol fed rats (**b**, **d**, **f**) were cultured on Transwell permeable supports for 5 days and then either untreated (un), or incubated with 10 μM control peptide (con; Ac-LYQY-NH_2_) or a claudin-5 extracellular domain mimetic peptide (C5; Ac-EFYDP-NH_2_) for 16 h. The cells were examined for barrier function by transepithelial resistance (TER) (**a**,**b**) and paracellular flux of calcein (**c**,**d**) and 10 kDa Texas Red dextran (**e**,**f**). The C5 peptide had little effect on barrier function of control AECs (**a**,**c**,**e**) however, it significantly increased TER (*P=0.014 versus untreated; ^#^*P*=0.042 versus control; *n*=6, one way ANOVA with Tukey multiple comparisons test) (**b**), and decreased paracellular flux of calcein (*P=0.007 versus untreated; ^#^*P*=0.054 versus control; *n*=3, one way ANOVA with Tukey multiple comparisons test) (**d**), and Texas Red Dextran (*P=0.009 versus untreated; ^#^*P*=0.040 versus control; *n*=3, one way ANOVA with Tukey multiple comparisons test) (**f**). (**g**) AECs as treated above were processed and examined by immunofluorescence for claudin-18 localization. Bar, 20 μm. Cells from alcohol fed rats showed a decrease in tight junction spikes, that was significantly less than that of untreated controls and alcoholic AECs that were either untreated or treated with a control peptide (*P<0.001 versus untreated; ^#^*P*<0.001 versus control peptide; ^†^*P*=0.041 versus untreated control AECs, *n*=9–11 fields from two independent experiments, one way ANOVA with Tukey multiple comparisons test) (**h**). Claudin-5 immunofluorescence is shown in Supplementary Fig. 11. (**i**–**l**) AECs as treated above were processed and examined by immunoblot for claudin-5, claudin-18 and ZO-1. Cells from alcohol-fed rats that were treated with the C5 peptide showed a significant and specific decrease in claudin-5 (*P=0.042 versus untreated; ^#^*P*=0.016 versus control; *n*=9, one way ANOVA with Tukey multiple comparisons test) (**l**). All quantitative data represents average±s.e.m.

As shown in Fig. 7b,d,f, overnight incubation of alcohol-exposed AECs with the Ac-EFYDP-NH_2_ peptide increased barrier function, as measured by an increase in TER and decrease in paracellular flux of calcein and Texas Red Dextran. By contrast, control AECs were unaffected by the Ac-EFYDP-NH_2_ peptide (Fig. 7a,c,e). A control peptide, Ac-LYQY-NH_2_, had no effect on AEC barrier function in either control or alcohol-exposed cells. The ability of Ac-EFYDP-NH_2_ to improve the barrier function of alcohol-exposed AECs correlated with a decrease in tight-junction spike formation (Fig. 7g,h) and a specific decrease in total claudin-5 content (Fig. 7j,l). Claudin-18 and ZO-1 were unaffected (Fig. 7i–l) as was claudin-1 (Supplementary Fig. 11e,f). These data provide an additional demonstration that an increase in endogenous claudin-5 diminishes AEC barrier function in response to alcohol and underscore the potential to directly target claudin-5 as a therapeutic approach to prevent alcoholic lung syndrome.

## Discussion

This study provides the first demonstration that an inter-claudin interaction has the capacity to affect claudin-scaffold protein interactions. Specifically, increased claudin-18:claudin-5 interactions decreased ZO-1:claudin-18 co-localization, which correlated with weakened assembly into tight junctions as evidenced by an increase in Triton X-100 solubility (Fig. 6). The net effect of decreased interactions between claudin-18 and ZO-1 is to destabilize tight junctions that, in turn, increases paracellular leak^34^. It is likely to be that claudin–claudin interactions beyond claudin-18:claudin-5 interactions will be found to play significant roles in the context of regulating assembly of claudins into tight junctions and in the organization of junctional scaffold complexes as signalling platforms that, in turn, affect paracellular permeability. Future work will determine whether or not this is the case.

Whether claudin-18:claudin-5 complexes are preformed or claudin-5 molecules newly delivered to the membrane destabilize claudin-18 is not known at present. Two examples of claudin–claudin interactions that occur before delivery to the plasma membrane are claudin-4:claudin-8 (ref. 35), and claudin-16:claudin-19 (ref. 36). In each of those cases, depletion or misfolding of one claudin resulted in intracellular accumulation of the other, evidence that these pairs of claudins serve as co-chaperones. Interestingly, in kidney epithelia, claudin-18 trafficking was independent of claudin-16 and claudin-19 (ref. 36), indicating specificity of *cis*–claudin interactions. In AECs, the intracellular pools of claudin-5 and claudin-18 are limited, largely vesicular and do not show complete co-localization. As the effects of claudin-5 on claudin-18 largely affect tight-junction morphology in AECs, and that these effects are antagonized by a claudin-5 extracellular mimetic peptide, it seems more likely to be that claudin-5 and claudin-18 interact within tight junctions or other regions of the plasma membrane rather than before delivery. Considering that tight-junction-associated claudins are highly dynamic^37^,^38^, there is certainly the capacity for claudin remodelling to occur within pre-formed tight junctions at cell–cell interfaces as well as in claudins newly delivered to the plasma membrane^39^.

Critically, this provides a novel mechanism for alcoholic lung syndrome whereby *cis* interactions between claudin-5 and claudin-18 can diminish barrier function by affecting the ability of claudin-18 to form complexes with ZO-1. *Cis* interactions between claudin-5 and claudin-3 have previously been characterized at a molecular level^32^,^40^, but this is the first demonstration that claudin-5 can regulate the ability of another claudin, in this case claudin-18, to interact with the cytoplasmic scaffold. Cytoplasmic scaffold proteins, including ZO-1 and ZO-2, have classically been thought of as being the primary regulators of claudin assembly into tight-junction strands by cross-linking claudins to the actin cytoskeleton^38^,^41^,^42^. In this model, claudins are essentially considered to be passive components that are directed by scaffold proteins such as ZO-1, to interact with actin and to sites were intercellular contacts can form^34^.

The ability of claudin–claudin interactions to regulate association of scaffold proteins with transmembrane components of tight junctions complements the classical model for scaffold protein–claudin interactions in which ZO-1 binds to the extreme carboxy-terminal domain of nearly all claudins and promotes interactions with the actin cytoskeleton. The hypothesis that claudin–claudin interactions can affect how the C-terminal tail interacts with the scaffold suggests that adjacent or co-heteroligomerized claudins have the capacity to attain conformations that either permit or restrict interactions with scaffold proteins. Although current high-resolution structural models of claudins have provided some insights into how claudins pack and form paracellular ion channels^31^,^43^, the C terminus is relatively unstructured and therefore how claudin–claudin interactions can affect its conformation are not known. From the gap junction literature, there are several examples where C termini of connexins in heteromeric channels regulates their conformation and channel function^44^,^45^. Although it remains to be determined, as ZO-1 interacts with the extreme terminal PDZ-binding motif of most claudins, interactions with ZO-1 are unlikely to occur unless the C terminus is fully extended and not sterically hindered.

Claudin-5 increased formation of tight-junction spikes that, in turn, correlated with increased paracellular leak. Association of tight-junction spikes with increased paracellular permeability is consistent with previous studies demonstrating that spikes and barrier dysfunction are also induced by transforming growth factor-β^4^ and nuclear factor-κB inhibitors^5^. In fact, normal AECs treated with the nuclear factor-κB inhibitor BMS-345541 showed both increased claudin-5 expression and increased formation of tight-junction spikes as a result of interfering with granulocyte–macrophage colony-stimulating factor signalling that mimics the effects of alcohol on AECs^5^. Here, live-cell imaging was used to confirm that these were sites where claudin-containing vesicles were observed to bud and fuse from the ends of spikes. Linking tight-junction spikes and enhanced endocytosis with a decrease in barrier function is also consistent with our previous demonstration that treatment of fetal AECs with endocytosis inhibitors almost doubled TER^46^, as well as with studies by other researchers demonstrating that increased junction protein endocytosis is associated with epithelial barrier dysfunction^47^,^48^,^49^,^50^.

Structures comparable to the tight-junction spikes observed here are also associated with keratinocyte desmosomal endocytosis induced by *Pemphigus vulgaris* antisera^51^, suggesting that spikes may be a general feature of squamous epithelial cells representing sites of active vesicle traffic involving deposition and internalization of junction proteins. It is also possible that spikes are sites where vesicle traffic is more readily visualized, and that vesicle budding and fusion occur at other locations in tight junctions, although the correlation between spike number and barrier dysfunction would argue against this possibility. In addition, whether spikes are formed by cuboidal epithelia remains to be determined and probably will require high-resolution three-dimensional imaging.

Given that tight-junction spikes are associated with alcohol and claudin-5 expression, and that these are sites of active vesicle trafficking of claudin-containing vesicles, our data demonstrate that increased claudin-5 is both necessary and sufficient to account for the deleterious effects of dietary alcohol on AEC barrier function. Although the effects of increased claudin-5 appear to contradict the role of claudin-5 in promoting endothelial barrier function^32^,^33^, our data demonstrate that claudin-5 function is cell type dependent and is influenced by the context of expression. For example, claudin-5 has the capacity to increase barrier function of MDCK II cells, which are otherwise exceptionally leaky, with baseline TER in the range of 100 Ω × cm^2^ (ref. 52). In AECs, which are much tighter, claudin-5 had the opposite effect. It is also possible that the ability of claudin-5 to impair tight junctions is specifically dependent on an interaction with claudin-18, which is not present in MDCK cells. A specific interaction between claudin-5 and claudin-18 has particular relevance to alveolar barrier function. Although increased claudin-5 was associated with alcoholic lung disease, the mechanism by which alcohol induces claudin-5 expression is under investigation at present and could either be transcriptional or posttranslational.

As claudin-5 has a dramatic effect on AEC barrier function, it represents an appealing potential pharmacologic target to improve alveolar barrier function in vulnerable individuals. Using a claudin-5 mimetic peptide (Ac-EFYDP-NH_2_) designed according to Baumgartner *et al.*^27^ we confirmed the feasibility of this approach, as this peptide specifically increased barrier function of alcohol-exposed AECs (Fig. 7a–f). We used an Ac-EFYDP-NH_2_ composed of D-amino acids, as Baumgartner *et al.*^27^ demonstrated that the D-amino acid version of an Ac-DFYNP-NH_2_ mimetic is 10–100-fold more effective than the corresponding L-amino acid version^27^. Unlike the DFYNP sequence that is shared by several claudins important for lung barrier function, including claudin-3 and claudin-4, the EFYDP corresponding to claudin-5 is unlikely to cross-react with other non-homologous claudins and claudin-1 expression in the lung is low and unaffected by the peptide (Supplementary Fig. 11e,f). Whether this level of specificity is sufficient to promote alveolar barrier function *in vivo* remains to be determined.

EFYDP is in the E2 region of the protein directly adjacent to the TM3 domain, a region of claudin-5 that mediates *cis*–claudin interactions^32^,^33^,^53^, consistent with our model that claudin-5 interactions with claudin-18 have a deleterious effect on the ability of claudin-18 to interact with ZO-1. The ability of a *cis*–claudin interaction to affect interactions of another claudin with the tight-junction scaffold represents a novel mode of tight-junction regulation with the potential to be pharmacologically manipulable. Specific and direct targeting of claudin-5 using these approaches offers the potential of preventing acute respiratory distress syndrome, particularly in those individuals at greatest risk due to underlying alcohol abuse, by improving alveolar barrier function and fluid clearance.

## Methods

### Cell culture

Animal protocols were reviewed and approved by the Institutional Animal Care and Use Committee of Emory University. Adult male Sprague–Dawley rats were pair-fed ethanol (36% of total calories) or control isocaloric maltin-dextrin using the liquid Lieber DeCarli Diet (Research Diets, New Brunswick, NJ) *ad libitum* for 6–8 weeks^4^. Animal use was limited to their use as a source for primary cells and thus sample size and randomization are not relevant variables.

Type II AECs were isolated from rats fed either alcohol or a control diet according to Dobbs^54^ with modifications. To remove red blood cells, lungs were perfused *in situ* with solution II (5.5 mM Dextrose, 10 mM HEPES, 2 mM CaCl_2_,12.3 mM MgSO_4_, 5 mM KCl and 140 mM NaCl pH 7.4) at 37 °C. Lungs were then removed, lavaged with cold PBS then lavaged with cold solution I (5.5 mM Dextrose, 10 mM HEPES, 0.197 mM EGTA,12.3 mM MgSO_4_, 5 mM KCl and 140 mM NaCl pH 7.4). Elastase (103 units per 40 ml solution II) was instilled into the lungs, which were incubated for 30 min at 37 °C. The lungs were then manually diced and resuspended in 5 ml fetal bovine serum (FBS)+5 ml DNase solution (1 mg ml^−1^ in solution II). The cells suspension was incubated for 10 min at 37 °C under gentle rotation, sequentially filtered through a 100-μm and then a 40-μm cell strainer (BD Biosciences), then centrifuged at 150 *g* for 8 min at 4 °C. The cell pellet was resuspended in 10 ml DMEM media (Sigma) containing 0.25 μg ml^−1^ amphotericin B (ThermoFisher), 100 U ml^−1^ penicillin:10 mg ml^−1^ streptomycin (Sigma), then biopanned to remove macrophages in polystyrene bacteriological 100 mm Petri dishes pretreated with 1.5 mg rat IgG per dish for 1 h at 37 °C. Using this approach, preparations routinely contained >90–95% type II AECs.

To produce model type-I AECs, 7.5 × 10^5^ cells in DMEM+10% FBS were plated in 1.12 cm^2^ Transwell-permeable supports (Corning 3460) pre-coated with 250 μl of 20 μg ml^−1^ rat tail type-I collagen in PBS (Roche Diagnostics, Mannheim, Germany), conditions that support differentiation to a type-I-like phenotype^55^,^56^. Culture media on both the apical and basolateral wells were changed every other day and cells were used for experiments on day 6 or 7 after seeding.

### Virus production and infection

Adenovectors encoding for NH_2_-terminal enhanced YFP–claudin-3 and control enhanced green fluorescent protein (EGFP) were prepared as previously described^12^. YFP–claudin-5 complementary DNA was produced as previously described^57^, removed using KpnI and XbaI, and then ligated into pAdLox using standard molecular biological techniques. YFP–claudin-18 and untagged claudin-5 were cloned into pAdeasy-1. It is noteworthy that for all claudin constructs the YFP was located on the N terminus of the claudin. Adenovirus particles were packaged and amplified by ViraQuest Inc. (North Liberty, IA). YFP–Claudin-5/AdLox was packaged by the National Heart, Lung and Blood Institute Viral Vector Core at the University of Pittsburgh. Alternatively, pAdLox plasmids were packaged and amplified by infecting HEK AD293 cells cultured in DMEM containing 5% heat-inactivated FBS, 0.25 μg ml^−1^ amphotericin B and 100 U ml^−1^ penicillin:10 mg ml^−1^ streptomycin. Virus particles were purified by caesium chloride centrifugation followed by dialysis against PBS^58^.

Control and claudin-5-specific lentivector shRNAs (Supplementary Table 1) were cloned into a modified expression vector pFH1þU6-UG-W using NheI and PacI as described^59^. Lentiviral particles were produced by the Emory Neuroscience NINDS Viral Core Facility.

AECs cultured on Transwell-permeable supports were transduced 4 days after isolation with either adenovector or lentivectors by adding virus particles to both the apical and basal media. For adenovectors and lentivectors, transduction was done at a multiplicity of infection of 5 and analysed 48 h after transduction, unless otherwise stated. Analysis was done 48 h post transduction. For lentivectors, cell media were changed 24 h after transduction.

### Barrier function measurements

TER measurements of AECs cultured on Transwell-permeable supports in Ringer's saline buffer (150 mM NaCl, 2 mM CaCl_2_, 1 mM MgCl_2_, 10 mM glucose and 10 mM HEPES pH 7.4) was measured using an Ohmmeter (World Precision Instruments, Sarasota, FL). Paracellular dye permeability was assessed by simultaneous measurement two different-sized fluorescent dyes across the cell monolayer for 2 h at 37 °C^5^,^12^. Flux assays were performed in Ringer's saline containing 50 μg ml^−1^ Texas Red Dextran (10 kDa) (ThermoFisher) and 2 μg ml^−1^ Calcein (0.62 kDa) (ThermoFisher) in the apical chamber. The amount of fluorophore that diffused into the basal chamber was measured using a microplate reader (Biotek Winooski, VT).

### Biochemical analysis

After 6 days in culture, AECs on Transwell-permeable supports were washed 2 × with Dulbecco's phosphate buffered saline (DPBS) and incubated for 20 min in 50 μl RIPA buffer (Cell Signaling). Cells were scraped off and debris were pelleted by centrifugation for 10 min at 13.200 *g* at 4 °C. Protein concentration of the supernatant was determined by BCA assay (ThermoFisher Pierce #23225). Reducing SDS sample buffer (10% glycerol, 1.25% SDS, 50 mM Tris pH 6.7 and 8.3 mg ml^−1^ dithiothreitol) was added to the supernatant. Protein samples were heated for 10 min at 70 °C then resolved by SDS–PAGE using 4–15% Mini-PROTEAN TGX stain-free gradient SDS–polyacrylamide gels, transferred to polyvinylidene difluoride or nitrocellulose membranes (BioRad, Hercules, CA, USA) and immunostained using primary antibodies and secondary antibodies indicated in Supplementary Table 2. For band detection, either Clarity Western ECL Substrate (BioRad) was used and imaged with the ChemiDocTMXRS system (BioRad) or fluorescence imaging was used with the Odyssey Classic imager (LI-COR). Image analysis and quantification was done using Image Lab software (BioRad) or using Image studio (LI-COR). Relative protein quantification was relative to actin. LI-COR images of immunoblottings were pseudocoloured to greyscale images in the figures. Uncropped versions of immunoblottings shown in the main body of the text are in Supplementary Fig.12.

### Co-immunoprecipitation

AECs were isolated and 2.5 × 10^6^ cells per well were plated on six-well Transwell-permeable supports (Corning 3450) coated with 20 μg ml^−1^ rat tail collagen (Roche, Nutley, NJ) and cultured for 6 days as described above. Cells were washed 2 × with ice-cold DPBS containing Ca^2+^ and Mg^2+^ (DPBS++). Cells were scraped in DPBS++ containing protease inhibitor cocktail without EDTA (Roche) and centrifuged at 4 °C, 500 *g* for 8 min. Next, cells were resuspended in DPBS++ with protease inhibitor cocktail without EDTA (Roche) containing 0.1% (v/v) Triton X-100, sonicated 3 × for 1 s and incubated for 30 min on ice. Cell lysates were centrifuged at 500 *g* for 8 min at 4 °C to remove large aggregates.

Before use, protein A magnetic beads (Sure Beads; BioRad) for co-immunoprecipitation were washed 3 × in DPBS++ (100 μl beads per 1 ml) and then blocked with DPBS++ containing protease inhibitor cocktail, 0.25% BSA and 0.2% Gelatin for 1 h at 4 °C. The cell supernatant was then incubated with 100 μl blocked, unlabelled beads for 3 h at 4 °C, to remove nonspecific interacting proteins. Precleared supernatant then was mixed with bead/antibody complexes (100 μl beads labelled with 1 μg antibody for 15 min at 4 °C) and incubated overnight at 4 °C. The next day, beads were washed 3 × with DPBS++ containing protease inhibitor cocktail. Beads were resuspended in 1 × SDS–PAGE sample buffer, then incubated for 10 min at 70 °C to elute proteins bound to beads. Protein samples were analysed by SDS–PAGE and immunoblotting as described above.

### Triton-X solubility assay

Tight-junction proteins were assessed for changes to Triton X-100 extractability as described earlier^12^ with modifications. After 6 days in culture on Transwell-permeable supports, AECs were washed 2 × with ice-cold DPBS. After washing, four wells were combined and cells were scraped 2 × into ice-cold DPBS containing Protease inhibitor cocktail with EDTA (Roche). Cells were centrifuged for 8 min at 500 *g* at 4 °C, resuspended in DPBS with protease inhibitor cocktail (Roche) containing 0.1% (v/v) Triton X-100 and incubated for 30 min at 4 °C. Next, cells were centrifuged at 100,000 *g* for 30 min at 4 °C, to separate the lysate into Triton-soluble (supernatant) and -insoluble (pellet) fractions. The samples were equivalently diluted in SDS–PAGE sample buffer, heated for 10 min at 70 °C, then analysed by SDS–PAGE and immunoblotting as described above.

### Fluorescence microscopy

AECs were cultured for 6 days on Transwell-permeable supports, were washed 3 × with DPBS containing Ca^2+^ and Mg^2+^ (DPBS++), fixed with 1:1 methanol/acetone for 2 min at room temperature (RT) and then washed again 3 × with DPBS++. For permeabilization, cells were washed once with DPBS++ containing 0.5% Triton X-100, 3 × with DPBS++ containing 0.5%Triton X-100 and 5% normal goat serum for 5 min. Next, cells were labelled for 1 h in DPBS++ containing 5% normal goat serum for 1 h at RT containing primary antibodies (Supplementary Table 2). Before secondary antibody incubation, cells were washed 3 × with DPBS containing 5% goat serum for 5 min, respectively. Cells were then incubated for 1 h with Cy-2 and/or or Cy3-conjugated antibodies (Supplementary Table 2) in DPBS with 5% normal goat serum. Cells were washed 3 × with DPBS++ with 5% normal goat serum and another 3 × with DPBS++ before mounting in Mowiol (Kuraray, Houston, TX) under a glass coverslip. Fluorescence images were taken using an Olympus IX70 microscope with a U-MWIBA filter pack (BP460–490, DM505, BA515–550) or U-MNG filter pack (BP530–550, DM570, BA590–800). Minimum and maximum intensities were adjusted for images in parallel so that the intensity scale remained linear to maximize dynamic range.

For Dynasore experiments, 5.0 × 10^5^ AECs isolated from control- or alcohol-fed rats were plated on collagen-coated Transwells and cultured for 6 days. On day 6, the cells were washed once with serum-free media. Serum-free media containing 0.25% dimethylsulfoxide (DMSO) (vehicle control), 40, 80 or 160 μM (in 0.25% DMSO) was put into each well. Cells were incubated for 4 h. Afterwards, cells were washed twice with DPBS++ and fixed with 1 ml Methanol/Acetone solution for 2 min before being immunostained for claudin-18.

Tight-junction spike quantification was done using cells immunolabelled for claudin-18. Samples used for morphometric analysis were blinded. Cells containing three or more projections that were perpendicular to the orientation of the intercellular junction were considered to be cells containing tight-junction spikes that were scored and expressed as a percentage of the total number of cells in the field. In Fig. 2, for control versus alcohol-exposed cells: 11 fields from 2 independent experiments each; number of cells scored: 383 control AECs and 563 alcohol-exposed AECs. For EGFP versus YFP–claudin-5 transduced cells: 11 fields from 2 independent experiments each; number of cells scored: 615 EGFP-transduced AECs and 392 YFP–cldn-5-transduced AECs. For alcohol-exposed AECs transduced with claudin-5 shRNA: 5 fields each; number of cells scored: 294 control cells, 206 shRNA1-treated cells and 244 shRNA2-treated cells. In Fig. 3, for Dynasore-treated alcohol-exposed cells: 8 fields from 2 independent experiments each; number of cells scored: 826 control cells, 728 cells treated with 40 μM Dynasore, 752 cells treated with 80 μM Dynasore and 863 cells treated with 160 μM Dynasore. For Dynasore-treated control cells: 9 fields from 2 independent experiments each; number of cells scored: 341 control cells, 227 cells treated with 40 μM Dynasore, 228 cells treated with 80 μM Dynasore and 273 cells treated with 160 μM Dynasore. In Fig. 7, for peptide-treated cells: 9–10 fields from 2 independent experiments each; number of cells scored: 165 untreated control cells, 186 untreated alcohol-exposed cells, 207 control-treated alcohol-exposed cells, 253 peptide-treated alcohol-exposed cells.

For live-cell imaging, 7.5 × 10^5^ alcohol exposed AECs were plated on onto glass-bottom culture dishes (MatTek Corp., Ashland, MA P50G-1.5-14-F) coated with rat tail type-I collagen (20 μg ml^−1^) (Roche Diagnostics). On day 4, cells were transduced with adenovirus encoding for YFP–claudin-5 or YFP–claudin-18 with a multiplicity of infection of 5, respectively. Media was changed 24 h after transduction. After 48 h expression, live-cell imaging using a Nikon A1R confocal laser scanning microscope with temperature control/CO_2_ chamber stage (× 40 oil lens, numerical aperture 1.3) and autofocus control was performed. Imaging was performed in Phenol Red-free Optimem containing 10% FBS, 0.25 μg ml^−1^ amphotericin B (Life technologies), 100 U ml^−1^ penicillin and 10 mg ml^−1^ streptomycin (Sigma) at 37 °C and 5% CO_2_. Data were collected with NIS-Elements AR 4.0 software (Nikon, Melville, NY). Imaging was performed over a time period of 20 min with 30 s intervals. Pictures were taken in 1,024 × 1,024 pixels resolution (excitation 488 nm and emission 525 nm) with low-excitation laser power of 1.2%, to minimize photo bleaching. Images and movies were processed with Image J. Minimum and maximum intensities were adjusted for images in parallel so that the intensity scale remained linear to maximize dynamic range.

### Stochastic optical reconstruction microscopy

To analyse the co-localization and particle size of claudin-18, ZO-1 and claudin-5 within the cell membrane STORM was performed^15^,^16^. Double-labelled secondary antibodies were prepared using donkey anti-rabbit (Jackson Immuno Research 711-005-152) and donkey anti-mouse IgG (Jackson Immuno Research 715-005-151). Stock labelling reagents were Alexa 647 carboxylic acid succinimidyl ester (2 μg μl^−1^; ThermoFisher A30000), Cy2 bisreactive dye (2 μg μl^−1^; GE Healthcare PA22000) and Cy3 monoreactive dye (2 μg μl^−1^; GE Healthcare PA23001) in anhydrous DMSO. For donkey anti-rabbit IgG, 1.5 μl Cy2, 0.6 μl Alexa 647 and 6 μl 1 M NaHCO_3_ were added to 62.5 μg per 50 μl IgG and incubated at RT for 30 min. The sample was diluted to 200 μl with PBS, then filtered using a NAP-5 Sephadex G-25 DNA Grade column (GE Healthcare 17-0853-02), washed with 550 μl PBS and eluted with 300 μl PBS. Donkey anti-mouse IgG was labelled in a similar manner, using 1.5 μl Cy3 instead of Cy2. Antibodies were stored at 4 °C and used within 2 months of preparation.

For immunolabelling, AECs were prepared as described above and plated onto glass-bottom culture dishes (MatTek Corp., MA P50G-1.5-14-F) coated with rat tail type-I collagen (20 μg ml^−1^; Roche Diagnostics). After 6 days in culture, cell immunostaining of claudin-18, ZO-1 and claudin-5 was performed as described above with the following changes. After permeabilization, cells were treated with 0.1% NaBH_4_ for 10 min at RT. Secondary incubation were washed with 3 × 1 ml DPBS++ then fixed/permeabilized with 1 ml 1:2 methanol/acetone for 2 min at RT. The cells were washed 3 × with DPBS++, treated with 0.1% NaBH_4_ for 10 min at RT, washed 3 × with DPBS++, washed once with DPBS++ with 0.5% Triton X-100, then twice with DPBS++ containing 0.5% TX-100, 2% normal goat serum. The cells were then incubated with rabbit anti-claudin-18+mouse anti-claudin-5 or rabbit anti-claudin-18+mouse anti-ZO-1 in DPBS++ containing 2% normal goat serum for 1 h at RT on a rotator platform. After primary antibody incubation, cells were washed 3 × with DPBS++ containing 2% normal goat serum and then incubated with a 1:100 dilution of double-labelled secondary antibody mixed in DPBS++ for 30 min at RT on a rotator platform. The cells were then washed 2 × for 1 h with DPBS++ containing 2% normal goat serum and then 3 × with DPBS++. Samples were post fixed with 3% paraformaldehyde+0.1% glutaraldehyde for 10 min at RT and washed 3 × with DPBS++. For imaging, antibody-labelled cells were incubated in 1.4 ml mercaptoethylamine imaging buffer (0.7 mg ml^−1^ glucose oxidase, 42.5 μg ml^−1^ catalase, 100 mM cystamine, 8.9 mM NaCl and 8.9% glucose in 44.3 mM Tris-HCl pH 8.0).

The samples were imaged with a Nikon N-STORM system based on an Eclipse Ti inverted microscope with the Perfect Focus System, × 100, 1.49 oil-immersion objective and an Andor iXon DU897 electron multiplying CCD (charge-coupled device) camera. Data were collected and analysed with NIS-Elements Software. Samples were excited with 457, 561 and 647 nm laser lines. Data collection involved alternating cycles of lower-intensity 457- and 561-nm activation pulses and high-intensity 647-nm imaging for localization and deactivation. High-resolution STORM images were collected over 20–30 min. Data were corrected for stage drift and localization fitted to Gaussian distributions using NIS-Elements set at minimum height of 250 nm and CCD baseline of 220 nm. Single-labelled and unlabelled samples were collected using the same parameters, to ensure that there was a lack of nonspecific signal detection and minimal cross-talk between fluorescent channels (Supplementary Fig. 8).

To analyse the size distribution of clusters containing claudin-18, claudin-5 or ZO-1 in the membrane, STORM images were analysed using ImagePro 3.0. Objects that consisted of 10 contiguous pixels with a threshold intensity of >50/255 were considered as the minimum cluster size. Ten pixels corresponded to an area of 585 nm^2^. Co-localization between of double-labelled STORM images was analysed by using ImageJ software. Given that total claudin-5 changes dramatically when comparing cells from control- and alcohol-fed animals, we calculated the co-localization index as the amount of claudin-18 or ZO-1 co-localizing with claudin-5 as opposed to the opposite calculation, which would be much more sensitive to changes in total claudin-5. Co-localized area between the red and the green channel was identified using a co-localization plugin for ImageJ (http://rsb.info.nih.gov/ij/plugins/co-localization.html). Two pixels were considered co-localized when the respective threshold of each channel was >50 (out of a range of 0–255) and the intensity ratio of the red and the green channel was >50%. Co-localized area as well as the area in the red and the green channel were quantified by using the particle analyser.

### Proximity ligation assay

For the PLA assay, AECs were cultured on Transwell-permeable supports for 6 days. On day 6, cells were washed 3 × with DPBS with Ca^2+^/Mg^2+^ and fixed with freshly made Methanol/Acetone (1:2) for 2 min. After fixation, the cells were washed 3 × with DPBS++, permeabilized with DPBS++ containing 0.5% (v/v) Triton X-100 for 5 min, then blocked with DPBS++ containing 0.5% (v/v) Triton X-100 and 5% goat serum (Sigma-Aldrich), and washed 2 × for 5 min under gentle agitation. Cells were incubated overnight in 250 μl DPBS++ containing 5% goat serum with primary antibody pairs (mouse anti-claudin-5+rabbit anti-claudin-18, rabbit anti-claudin-5+mouse anti-ZO-1 or rabbit anti-claudin-18+mouse anti-ZO-1; Supplementary Table 2). The next day, filters were washed 3 × with DPBS++ containing 5% goat serum (v/v) for 5 min under gentle agitation. After washing, the Transwell filters were cut out and put upside down on Parafilm and a 50-μl solution containing the secondary antibodies (anti-rabbit Plus (DUO92002) and anti-mouse Minus (DUO92004) diluted in DPBS++ containing 5% goat serum (v/v)) was pipetted under the filter. Filters were incubated for 1 h in a humidified incubator at 37 °C, 5% CO_2_. For the detection of protein–protein interactions, the detection Kit Red (DUO92008) was used according to the manufacturer's instructions. Filters were mounted on slides using Duolink In Situ Mounting Medium with 4,6-diamidino-2-phenylindole (DUO82040), covered with glass cover slips, sealed with nail polish and stored at −20 °C until imaging. Fluorescence images were taken using an Olympus IX70 microscope with a U-MWIBA filter pack (BP460–490, DM505, BA515–550) or U-MNG filter pack (BP530–550, DM570, BA590–800). Minimum and maximum intensities were adjusted for images in parallel so that the intensity scale remained linear to maximize dynamic range. Image analysis was performed using the Fiji particle analyser tool. PLA signal intensity was analysed by measuring the number of individual clusters above a threshold intensity value of 70.

### Claudin-5 mimetic peptide treatment

To antagonize the deleterious effects of claudin-5 on barrier function in alcohol-exposed AECs, a short D-peptide targeting claudin-5 was designed (Ac-EFYDP-NH_2_; Ac, Acetylation, NH_2_, amide) analogous to the claudin-3/4 peptide that was previously described^27^ and synthesized by LifeTein (Sumerset, NJ). An Ac-LYQY-NH_2_ peptide was also synthesized and used as peptide control^27^. Peptides were dissolved in 30% DMSO in water at a concentration of 30 mM (30,000 × stock). AECs from either control- or alcohol-fed rats were cultured for 5 days on Transwell-permeable supports and then the apical medium was replaced with 500 μl DMEM media containing 10 μM peptide (final DMSO concentration 0.01%). DMSO alone was used for untreated controls. AECs were incubated for 16 h and then assessed for barrier function, immunofluorescence or immunoblotting as described above.

### Statistics

All statistics were calculated using GraphPad Prism 6.0. Statistical significance for parametric data was determined using unpaired two-tailed *t*-test to compare one dependent variable against one independent variable, one-way analysis of variance with Tukey's multiple comparisons test to compare one dependent variable against multiple independent variables and two-way analysis of variance with Bonferroni multiple comparisons test to compare multiple dependent variables against multiple independent variables and non-parametric data using the Mann–Whitney *U*-test. Sample size was determined so that we could detect a minimum 20% difference in values with s.e. of±10%. Variance between compared groups was comparable throughout the study. Data in most graphs represent average±s.e., box and whisker plots in Supplementary Fig. 7g–i show the median value, 25th and 75 percentiles as the limits of the box and 5th and 95th percentiles as the limits of the whiskers.

### Data availability

The source data that support the findings of this study are available from the corresponding author (MK) upon request.

## Additional Information

**How to cite this article:** Schlingmann, B. *et al.* Regulation of claudin/zonula occludens-1 complexes by hetero-claudin interactions. *Nat. Commun.* 7:12276 doi: 10.1038/ncomms12276 (2016).

## Supplementary Material

Supplementary Information Supplementary Figures 1-12, Supplementary Tables 1-2

Supplementary Movie 1 YFP-cldn-5 containing vesicles bud from and fuse with tight junction spikes in alveolar epithelial cells. Live cell imaging was performed with alcohol exposed AECs transduced with Adenovirus encoding YFP-claudin-5.Videos were acquired over a 20 minute time period with a frame capture of 30 second intervals. Labeled vesicles containing YFPclaudin-5 (arrow) were found to both fuse to and bud from tight junction spikes, demonstrating that these are dynamic structures.

Supplementary Movie 2 YFP-cldn-18 containing vesicles bud from and fuse with tight junction spikes in alveolar epithelial cells. Live cell imaging was performed with alcohol exposed AECs transduced with Adenovirus encoding YFP-claudin-18.Videos were acquired over a 20 minute time period with a frame capture of 30 second intervals. Labeled vesicles containing YFPclaudin-18 (arrow) were found to both fuse to and bud from tight junction spikes, demonstrating that these are dynamic structures.
