# Supplementary material for: Regulation of claudin/zonula occludens-1 complexes by hetero-claudin interactions
Source: Nat Commun. 2016 Jul 25;7:12276. doi: 10.1038/ncomms12276 (PMC4962485; doi:10.1038/ncomms12276)
Supplement: Supplementary Information — Supplementary Figures 1-12, Supplementary Tables 1-2 [file ncomms12276-s1.pdf]

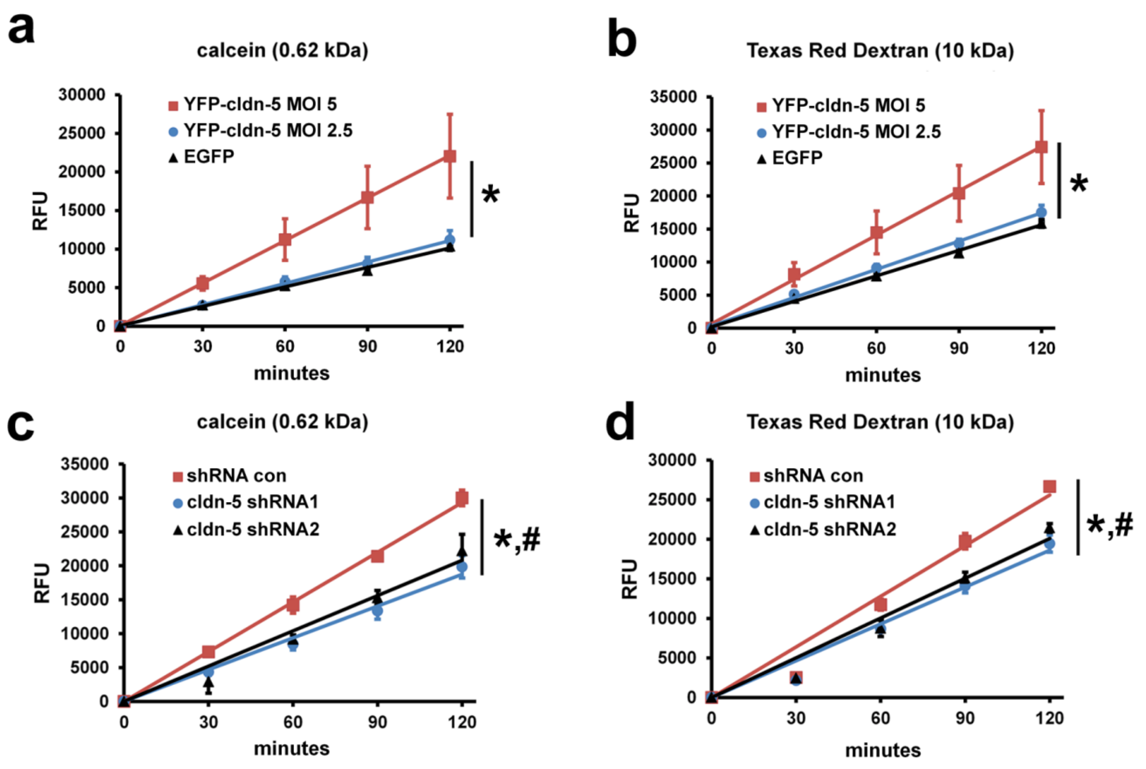

Supplementary Figure 1: Increased claudin-5 correlates with increased paracellular flux. **(a,b)** AECs isolated from control fed rats were cultured for 6 days on Transwell permeable supports. On day 4 AECs were transduced with adenovirus encoding YFP-claudin-5 with an MOI of 2.5 or 5. AECs transduced with adenovirus encoding for EGFP were used as controls (MOI 5). 48h after transduction, the cells were assessed for paracellular dye flux using calcein (n=4) **(a)** and 10 kDa Texas Red Dextran (n=4) **(b)**. AECs expressing YFP-claudin-5 showed increased dye permeability (* - p = 0.002 **(a)**; p = 0.017**(b)**, two way ANOVA with Bonferroni multiple comparisons test). **(c,d)** AECs from alcohol fed rats were treated with lentiviruses containing shRNA targeting claudin-5 (cld-5 shRNA1; shRNA2) or control scrambled shRNA (con) prior to culture on Transwell permeabile supports. On day 6, the cells were assessed for paracellular dye flux. Paracellular flux of calcein **(c)** and Texas Red Dextran **(d)** was significantly decreased by treatment with cldn-5 shRNA. For calcein **(c)**, * - p=007 shRNA1 vs control; # - p = 0.044 shRNA2 vs. control (n=3, two way ANOVA with Bonferroni multiple comparisons test). For Texas Red Dextran **(d)**, * - p=004 shRNA1 vs control; # - p = 0.002 shRNA2 vs. control (n=3, two way ANOVA with Bonferroni multiple comparisons test). All quantitative data represents average ± SEM.

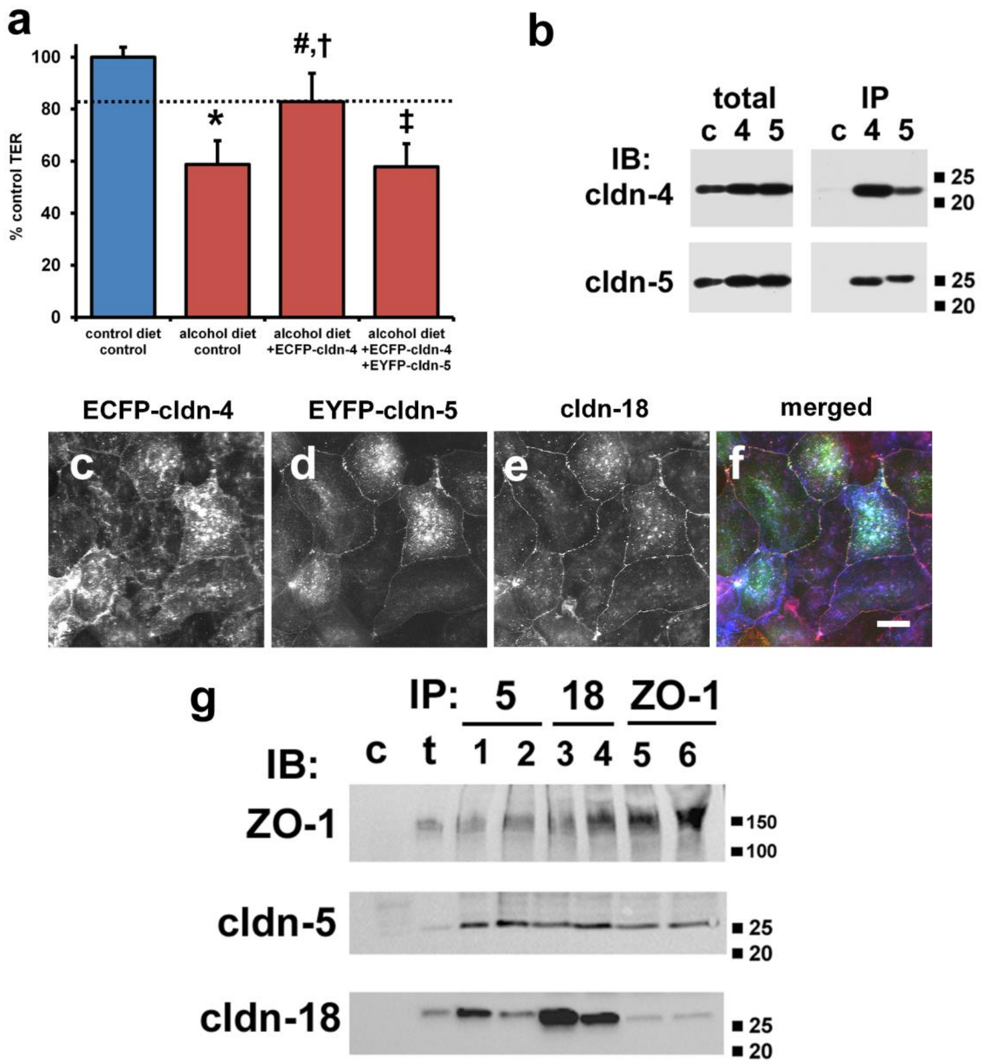

Supplementary Figure 2: Claudin-5 interacts with claudin-4, claudin-18 and ZO-1. (a) To test the effects of increasing claudin-4 expression on AEC barrier function, alcohol- exposed AECs were transduced with either EGFP (control), ECFP-claudin-4(MOI 5) alone or ECFP-claudin-4 (MOI 5) + YFP-claudin-5 (MOI 5) 48h after transduction, TER was measured **(a)**. Alcohol-exposed AECs had significantly lower TER than controls (n=6, * - $p < 0.0001$, one way ANOVA with Tukey multiple comparisons test). Increasing claudin-4 expression significantly increased TER of alcohol exposed AECs relative to control alcohol cells (n=6, # - p=0.016, one way ANOVA with Tukey multiple comparisons test), although the level of recovery did not match control cell levels (n=6, † - p=0.012, one way ANOVA with Tukey multiple comparisons test). Moreover, co-transduction with YFP-claudin-5 antagonized the ability of ECFP-claudin-4 to improve barrier function (n=6, ‡ - p=0.013, one way ANOVA with Tukey multiple comparisons test). Data represents average ± SE. **(b)** AECs cultured on Transwell permeable supports were lysed in 0.1% Triton X-100 and assessed for the ability of claudin-4 and claudin-5 to co-immunoprecipitate (IP) as determined by immunoblot (IB). Total lysates are shown in the left panel. Protein A agarose beads without primary antibody that were incubated with

lysate was used as a nonspecific control (c). There was reciprocal co-IP between claudin-5 and claudin-4. **(c-f)**. Alcohol- exposed AECs transduced with ECFP-claudin-4 **(c)** + YFP-claudin-5 **(d)** were fixed, permeabilized and processed for claudin-18 (e) immunofluorescence. A merged image containing all three channels is in **(f)**. Scale bar - 10µm. **(g)** AECs cultured on Transwell permeable supports were lysed in 0.1% Triton X-100 and assessed for the ability of claudin-5, claudin-18 and ZO-1 to co-immunoprecipitate (IP) as determined by immunoblot (IB). Protein A agarose beads without primary antibody that were incubated with lysate was used as a nonspecific control (c), total cell lysate (t) is also shown. Antibodies for IP are 1: mouse anti-cldn-5; 2: rabbit anti-cldn-5; 3: rabbit monoclonal anti-claudin-18; 4: rabbit anti-claudin-18 (mid); 5: rabbit anti-ZO-1; 6: rabbit anti-N-term-ZO-1. Claudin-5, claudin-18 and ZO-1 in the Triton X-100 soluble fraction interacted to varying extents. This provides biochemical evidence that these tight junction proteins interact in native AECs. All quantitative data represents average ± SEM.

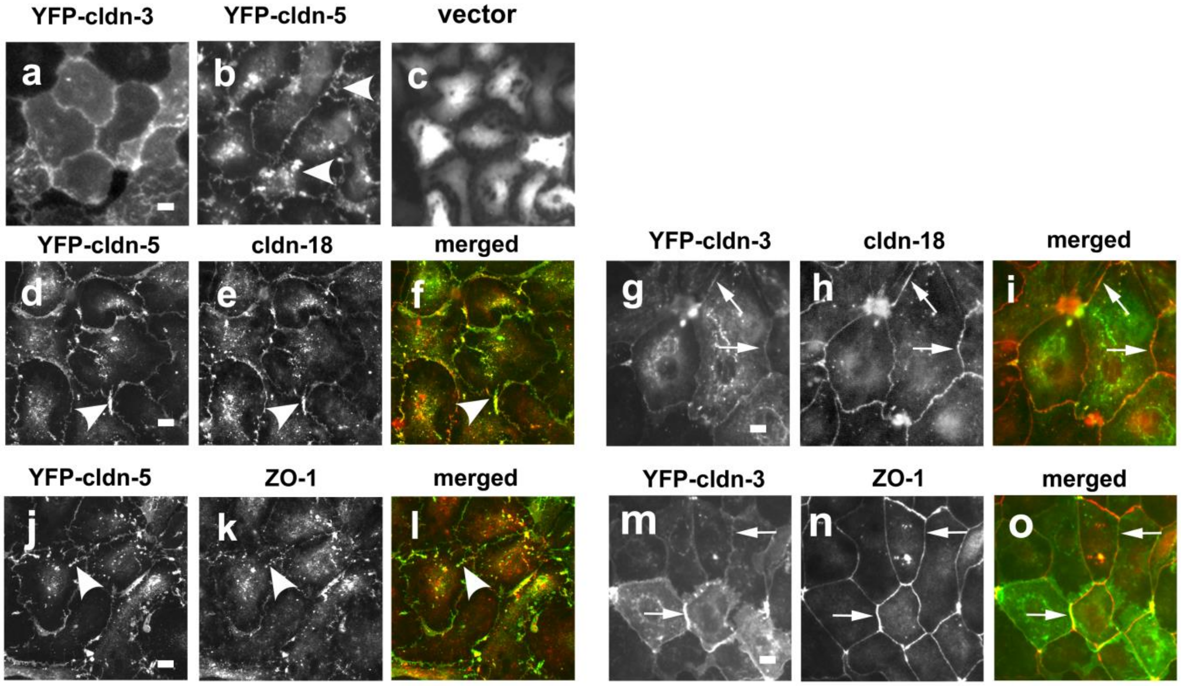

Supplementary Figure 3: Increased formation of tight junction spikes is specific for increased claudin-5. Representative images of normal AECs transduced with adenovirus encoding YFP-claudin-3 **(a,g-i,m-o)**, YFP-claudin-5 **(b,d-f,j-l)** or EGFP as control **(c)**. Images of live cells expressing YFP-claudin-3 showed normal tight junction morphology **(a)** as opposed to live cells expressing YFP-claudin-5 **(b).** Arrowheads indicate areas where tight junction spikes were formed. AECs were fixed, permeabilized and immunolabeled for claudin-18 or ZO-1 in cells expressing YFP-claudin-5 **(d-f,j-l)** and YFP-claudin-3 **(g-i,m-o)**. Localization of claudin-18 and ZO-1 was disrupted by YFP-claudin-5 expression **(arrowheads, d-f,j-l)**. By contrast, claudin-18 and ZO-1 in AECs expressing YFP-claudin-3 were not disrupted, suggesting that the formation of tight junction spikes was specific for increased claudin-5 expression. Arrows show regions where YFP-cldn-3 co-localized with claudin-18 and ZO-1. Scale bar - 10µm

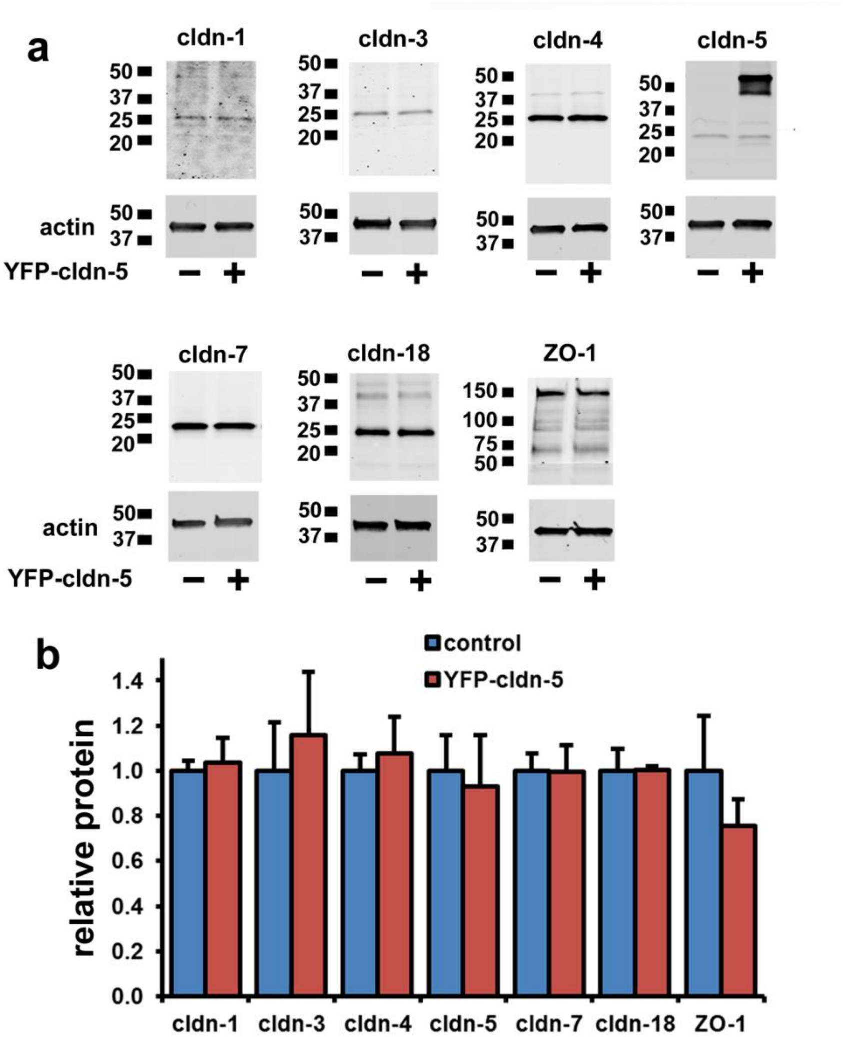

Supplementary Figure 4: YFP-claudin-5 has no effect on expression of endogenous tight junction proteins. (a,b) Normal AECs on Transwell permeable supports that were either untreated (-) or transduced with adenovirus encoding YFP-claudin-5 (+) were processed and analyzed by immunoblot for claudin-1, claudin-3, claudin-4, claudin-5, claudin-7, claudin-18, ZO-1 and actin. By densitometry, there was not a significant effect of YFP-cldn-5 expression on any of the tight junction proteins examined **(b)** (n=3, average $\pm$ SEM, unpaired two-tailed t-test).

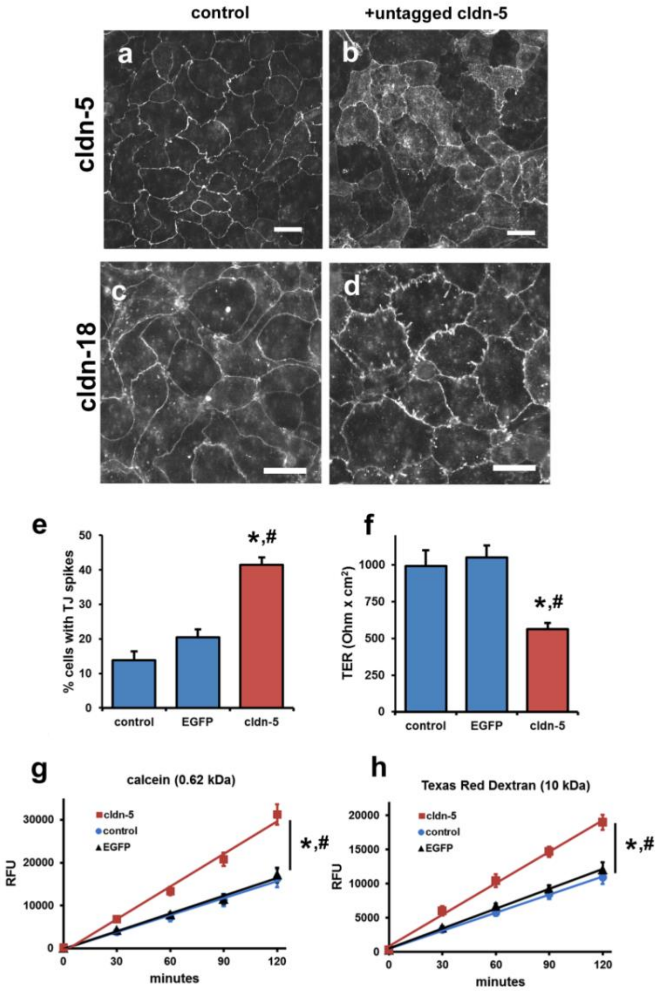

Supplementary Figure 5: Increased expression of untagged claudin-5 induces formation of tight junction spikes and impairs alveolar barrier function. (a-d) Normal AECs on Transwell permeable supports that were either untreated controls **(a,c)** or transduced with adenovirus encoding untagged claudin-5 **(b,d)** were processed for immunofluorescence analysis of claudin-5 **(a,b)** or claudin-18 **(c,d)**. Bar – 20 µm. Cells transduced with claudin-5 showed a significant increase in tight junction spikes containing claudin-18 (* - $p < 0.001$ vs control; # - $p < 0.001$ vs EGFP transduced AECs, n=9 fields from two independent experiments, one way ANOVA with Tukey multiple comparisons test) **(e).** **(f-h)** AEC barrier function was decreased by increased expression of untagged claudin-5, as assessed by transepithelial resistance (TER) (* - $p = 0.0011$ vs control AECs; # - $p = 0.0002$ vs EGFP transduced AECs, n=6, one way ANOVA with Tukey multiple comparisons test) **(f)** and paracellular flux of calcein (* - $p < 0.0001$ vs control AECs; # - $p<0.0001$ vs EGFP transduced AECs, n=3, two way ANOVA with Bonferroni multiple comparisons test) **(g)** and Texas Red dextran (* - $p < 0.0001$ vs control AECs; # - $p = 0.0004$ vs EGFP transduced AECs, n=3, two way ANOVA with Bonferroni multiple comparisons test) **(h).** All quantitative data represents average ± SEM.

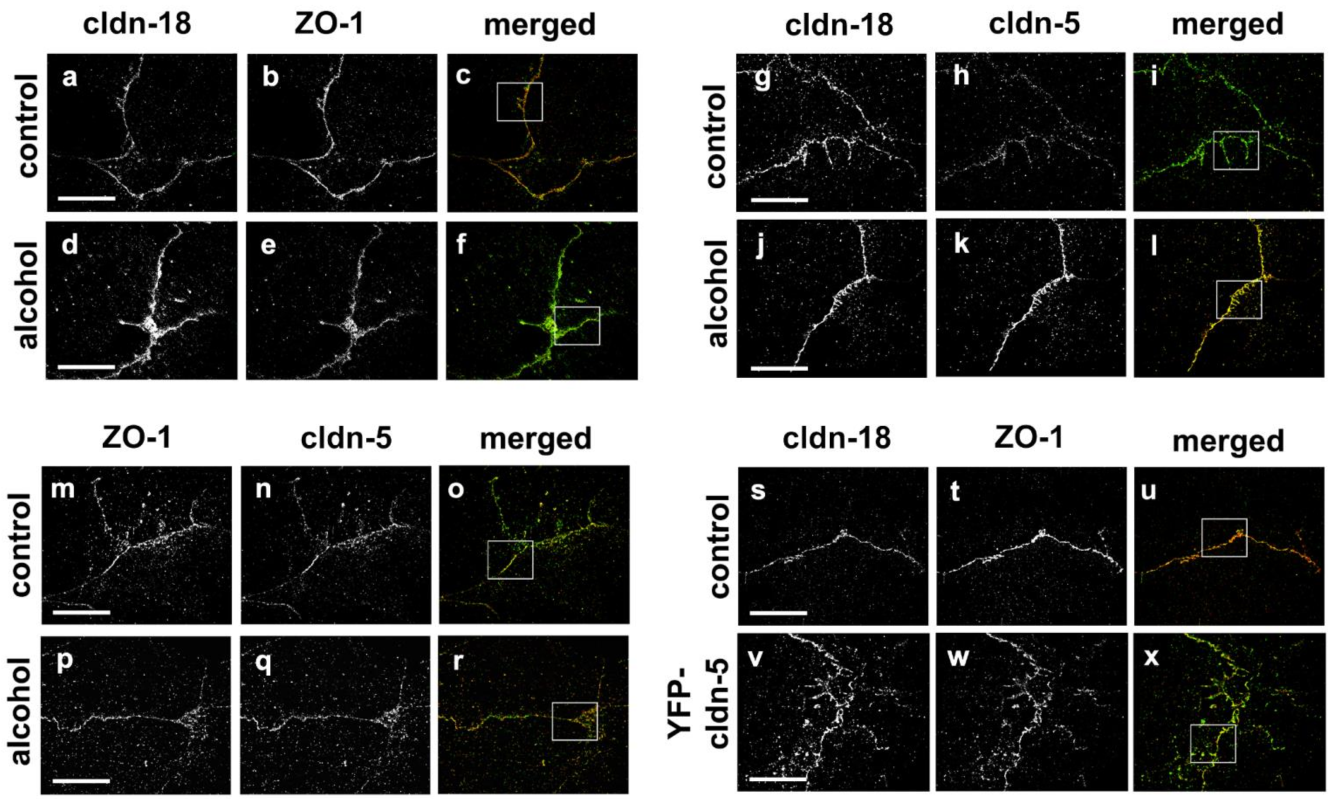

Supplementary Figure 6: Low magnification STORM images. (a-r) AECs isolated from alcohol fed rats and controls were cultured, processed and imaged by STORM as described in Methods. Images in **(a-r)** are low magnification images of the fields shown in Figure 4. (**s-x**) are low magnification images of the fields shown in Figure 5. In each case, the square region in each merged image represents the high magnification images shown in Figures 4 and 5. Cells were co-immunolabeled for either claudin-18 and ZO-1 **(a-f; s-x)**, claudin-5 and ZO-1 **(m-r)** or claudin-5 and claudin-18 **(g-l)**. Bar - 10µm.

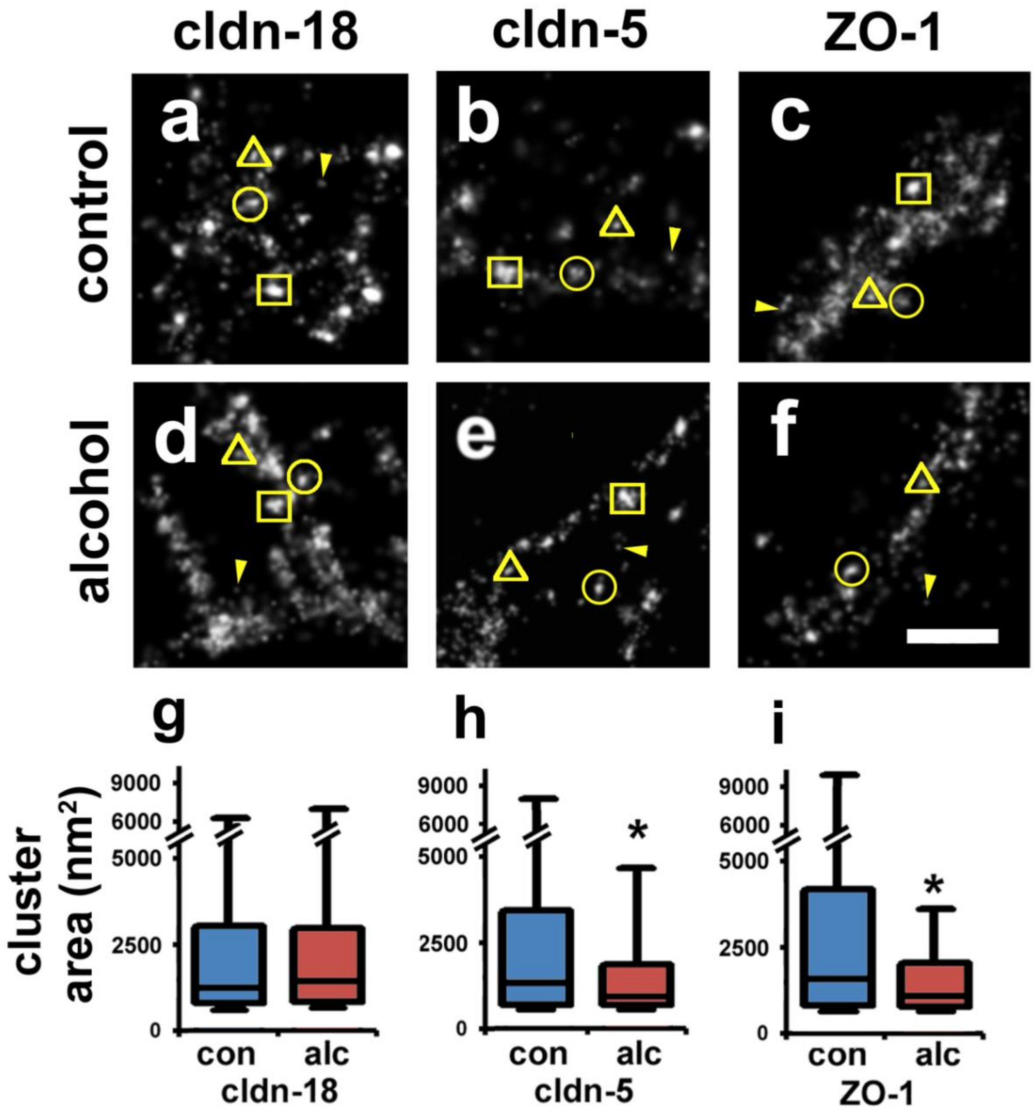

Supplementary Figure 7: Alcohol alters the organization of tight junction associated ZO-1 and claudin-5. AECs isolated from control **(a-c)** or alcohol fed rats **(d-f)** were processed, imaged by STORM and cluster size was determined by using Image Pro 3.0 as described in Methods. Examples of clusters with different diameter are labeled by the yellow square (90-100 nm), circle (65-85 nm), triangle (35-45 nm) and arrowhead (25 nm). Bar – 0.5 µm. In alcohol-exposed AECs the cluster size for claudin-18 **(g)** was unchanged. By contrast, cluster size for claudin-5 **(h)** and ZO-1 **(i)** was significantly decreased ($p < 0.01$, Mann Whitney U test). Data in (s-u) show box and whiskers plots of cluster size, where the line represents the median value, the limits of the box represent 25th and 75th percentiles and the limits of the whiskers represent 5th and 95th percentiles. Total number of clusters analyzed: claudin-18 control=826, alcohol=4009 **(g)**, claudin-5 control=1709, alcohol= 1594 **(h)** and ZO-1 control=3683, alcohol=5268 **(i)**.

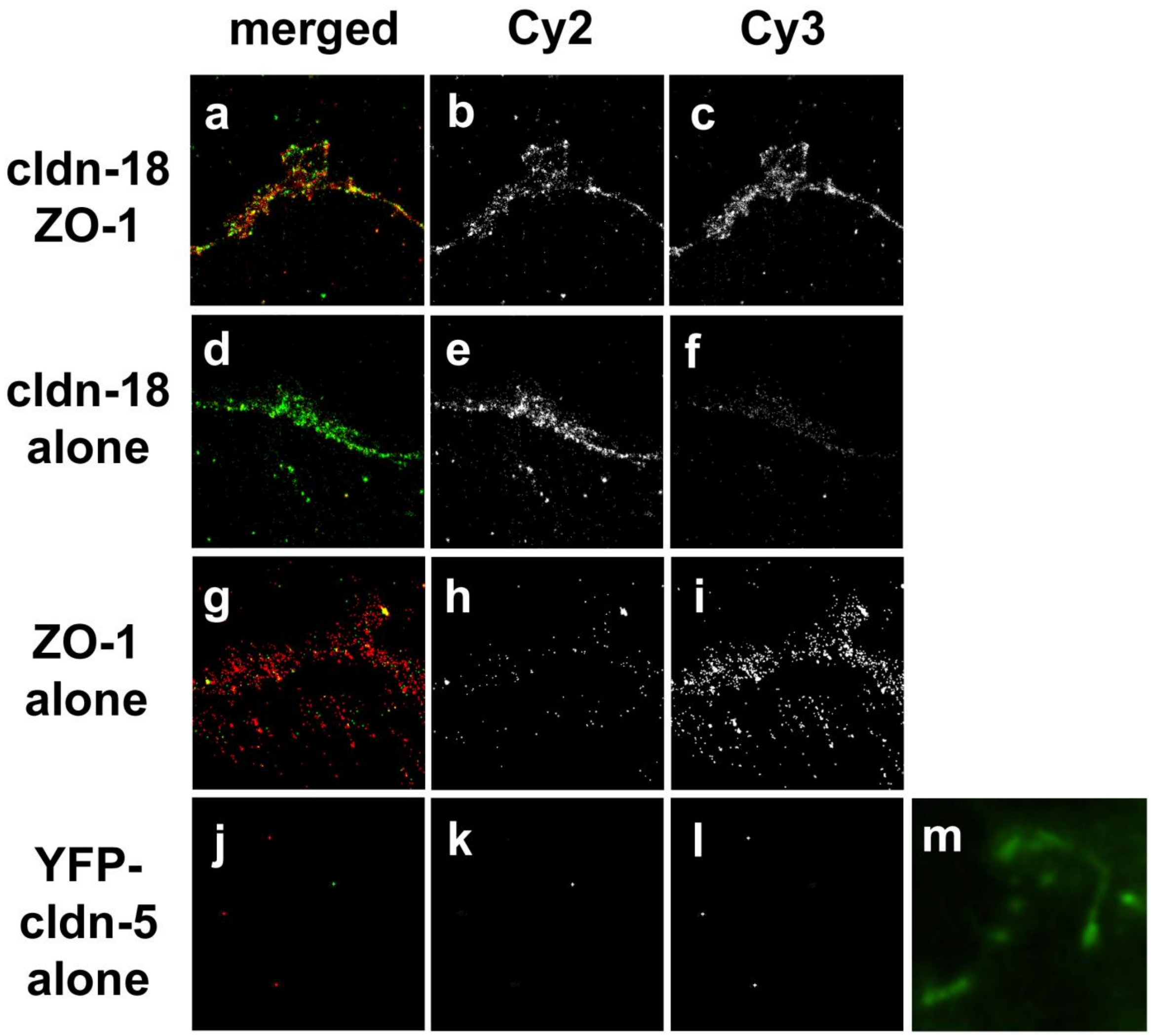

Supplementary Figure 8: Single labeled images to control for crosstalk in STORM. Normal AECs were isolated, cultured and processed for STORM. The AECs were immunolabeled for claudin-18 and ZO-1 **(a-c)**, claudin-18 alone **(d-f)**, ZO-1 alone **(g-i)** or were unlabeled, but transduced with YFP-claudin-5 **(j-m)**. Single labeled Claudin-18 and ZO-1 showed little cross talk in the complementary channel **(f,h)**. Moreover, unlabeled AECs expressing YFP-claudin-5 **(j-l)** were undetectable by STORM under the conditions and settings used, so that this construct could be used to measure the effects of increased claudin-5 expression on ZO-1 and claudin-18. **(m)** YFP-claudin-5 as imaged by conventional confocal fluorescence microscopy corresponding to the images in panels **(j-l)**.

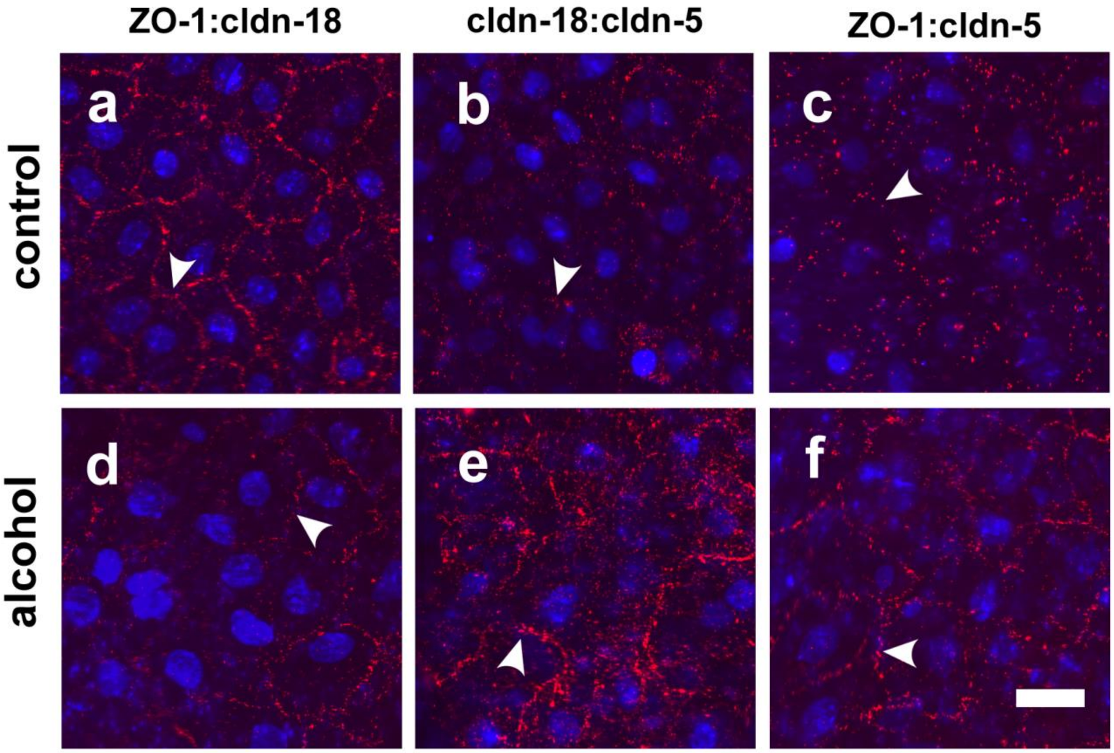

Supplementary Figure 9: Claudin-5 induced by alcohol decreases ZO-1:claudin-18 co-localization as determined by proximity ligation assay. AECs isolated from control **(a-c)** or alcohol **(d-f)** fed rats were cultured, immunolabeled and analyzed using the proximity ligation assay (PLA) as well as counterstained with DAPI to label nuclei. Cells were PLA-labeled for claudin-18 and ZO-1 **(a,d)**, claudin-5 and claudin-18 **(b,e)** or claudin-5 and ZO-1 **(c,f)**. Arrowheads denote PLA signals at sites corresponding to tight junctions. Bar, 20 µm.

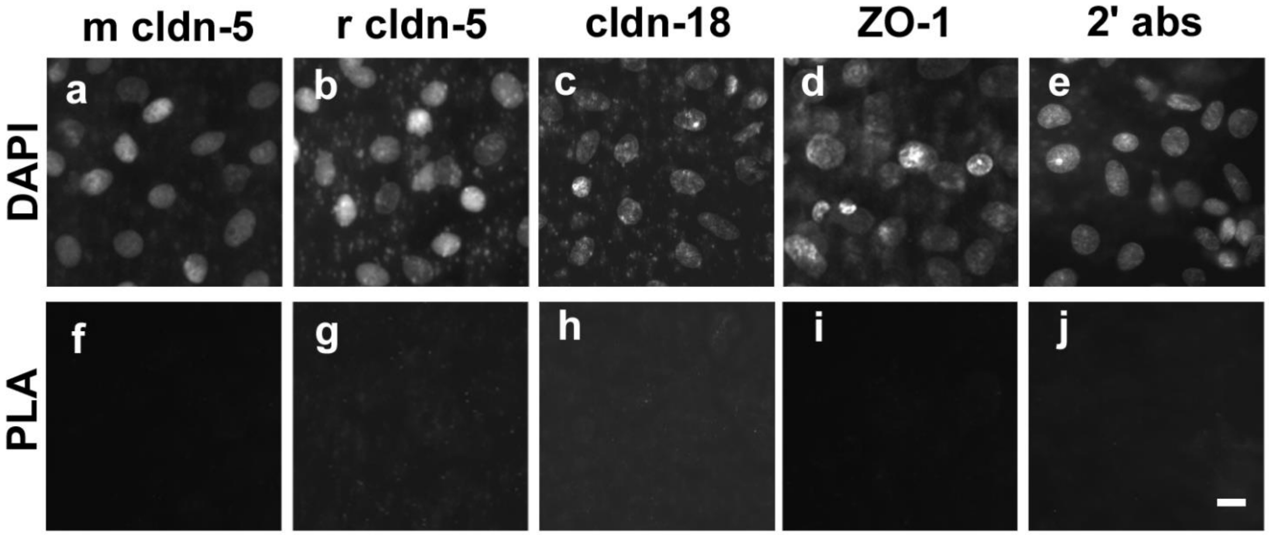

Supplementary Figure 10: Negative control images for the proximity ligation assay (PLA). AECs were single-labeled with mouse anti-claudin-5 **(a,f)**, rabbit anti-claudin **(b,g)**, rabbit-anti-claudin-18 **(c,h)**, rabbit anti-ZO-1 **(d,i)** and then processed for PLA as described in Methods. **(e,j)** cells were incubated with both PLA probe secondary antibodies alone and then processed for PLA. In all cases, samples were counterstained with DAPI to label nuclei **(a-e)**. In each case there was little if any PLA signal. Bar – 10 μm

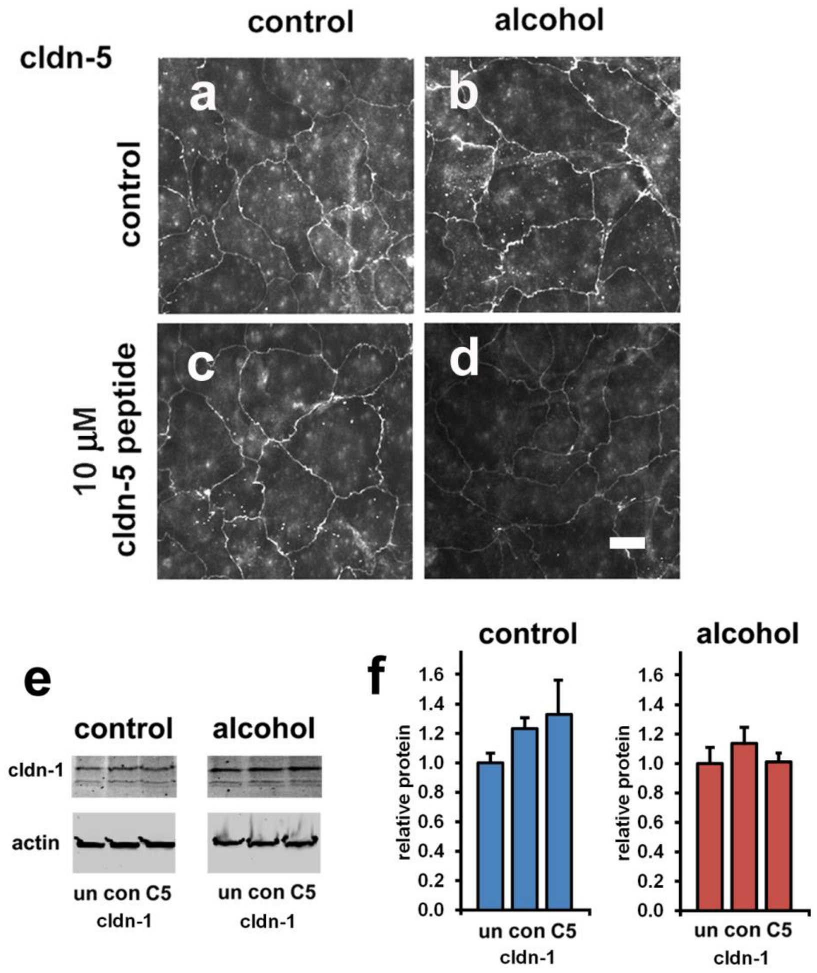

Supplementary Figure 11: Effect of a claudin-5 extracellular domain mimetic on claudin-5 localization. AECs from either control fed **(a,c)** or alcohol fed rats **(b,d)** were treated with either scrambled control peptide **(a,b)** or a claudin-5 specific peptide **(c,d)** at 10 µM for 16 h and then fixed and processed for claudin-5 immunofluorescence. Bar – 20 µm. **(e,f)** AECs as treated above were processed and examined by immunoblot for claudin-1. The C5 peptide had no effect on claudin-1 expression (one way ANOVA with Tukey multiple comparisons test). All quantitative data represents average ± SEM.

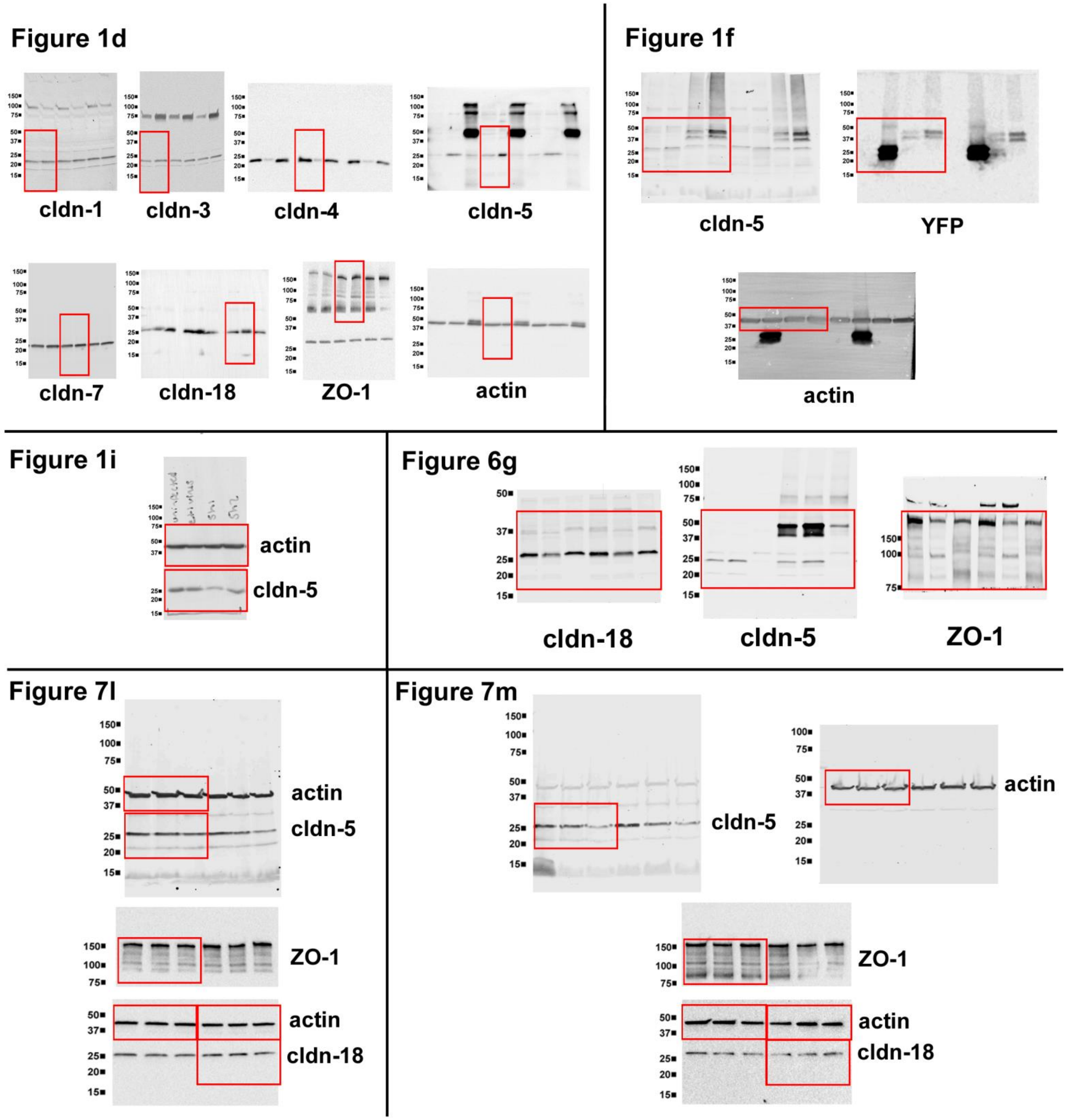

Supplementary Figure 12: Full scans of immunoblots presented in the main figures. Representations in main figures are the regions delineated by red rectangles.

Supplementary Table 1 – shRNA constructs used in this study

**Cldn-5 shRNA1**	**616-624**					
Sense	5' (NheI)	CCCC	CCAACGGCGATTACGACAA	TTCAAGAGA	TTGTCGTAATCGCCGTTGG	TTTTTGG (PacI) 3'
Anti	5' (PacI)	CCAAAAA	CCAACGGCGATTACGACAA	TCTCTTGAA	TTGTCGTAATCGCCGTTGG	GGGG (NheI) 3'
**Cldn-5 shRNA2**	**1594-1612**					
sense	5' (NheI)	CCCC	CCACCAAACTGCCGCTAAC	TTCAAGAGA	GTTAGCGGCAGTTTGGTGG	TTTTTGG (PacI) 3'
anti	5' (PacI)	CCAAAAA	CCACCAAACTGCCGCTAAC	TCTCTTGAA	GTTAGCGGCAGTTTGGTGG	GGGG (NheI) 3'
**scrambled**						
sense	5' (NheI)	CCCC	AGTCATTGACGACAGCGTA	TTCAAGAGA	TACGCTGTCGTCAATGACT	TTTTTGG (PacI) 3'
anti	5' (PacI)	CCAAAAA	AGTCATTGACGACAGCGTA	TCTCTTGAA	TACGCTGTCGTCAATGACT	GGGG (NheI) 3'

Supplementary Table 2 – Antibodies used in this study

Name	Company	Catalog #	IB dilution	IF dilution
Rabbit anti-claudin-1	Thermo Fisher	519000	1:500	
Rabbit anti-claudin-3	Thermo Fisher	341700	1:500	1:150
Rabbit anti-claudin-4*	Thermo Fisher	364800	1:500	
Mouse anti-claudin-4	Thermo Fisher	329400		1:150
Rabbit anti-claudin-5*	Thermo Fisher	341600	1:1000	1:100
Mouse anti-claudin-5*	Thermo Fisher	35200	1:1500	1:150
Rabbit anti-claudin-7	Thermo Fisher	349100	1:500	
Rabbit anti-claudin-18 monoclonal*	Thermo Fisher	700178	1:2000	1:125
Rabbit anti-claudin-18 mid-term*	Thermo Fisher	388100	1:2000	1:125
Mouse anti-ZO-1	Thermo Fisher	339100		1:100
Rabbit anti-ZO-1*	Thermo Fisher	617300	1:1500	
Rabbit anti-ZO-1 N-term*	Thermo Fisher	402300	1:1500	
Rabbit anti-GFP	Novus Biologicals	NB600-380	1:2500	
Mouse anti-GFP	Novus Biologicals	NB600-597	1:2500	
Rabbit anti-actin N-term	Sigma	A2103	1:10000	
Mouse anti-actin	Thermo Fisher	40MI	1:10000	
Mouse anti-ß-catenin	BD Biosciences	610153	1:2000	1:200
Goat anti-rabbit IgG IRDye 800CW	Licor	92632211	1:10000	
Goat anti-mouse IgG IRDye 680RD	Licor	92668070	1:10000	
Mouse anti-rabbit IgG conformation specific (L27A9) HRP	Cell Signalling	5127S	1:5000	
Horseradish peroxidase-conjugated goat anti-rabbit IgG	Jackson Immuno	111-035-144	1:10000	
Horseradish peroxidase-conjugated goat anti-mouse IgG	Jackson Immuno	115-035-166	1:10000	
Cy3 AfiniPure goat anti-mouse IgG	Jackson Immuno	115-165-1660		1:3000
Cy2 AffiniPure goat anti-rabbit IgG	Jackson Immuno	111-225-1440		1:2000
AffiniPure donkey anti-rabbit IgG[#]	Jackson Immuno	711-005-152		1:100
AffiniPure donkey anti-mouse IgG[#]	Jackson Immuno	715-005-151		1:100
PLA probe Plus Donkey anti-Rabbit IgG	Sigma-Aldrich	DUO92002		1:5
PLA probe Minus Donkey anti-mouse IgG	Sigma-Aldrich	DUO92004		1:5

IB – immunoblot, IF – immunofluorescence (including STORM and PLA).

* - Primary antibodies used for co-immunoprecipitation were pre-incubated with Protein A magnetic beads prior to use as described in Methods.

[#] - Secondary antibodies used for STORM were double-labeled prior to use as described in Methods.
